# Predicting in vivo effect levels for repeat-dose systemic toxicity using chemical, biological, kinetic and study covariates

**DOI:** 10.1007/s00204-017-2067-x

**Published:** 2017-10-27

**Authors:** Lisa Truong, Gladys Ouedraogo, LyLy Pham, Jacques Clouzeau, Sophie Loisel-Joubert, Delphine Blanchet, Hicham Noçairi, Woodrow Setzer, Richard Judson, Chris Grulke, Kamel Mansouri, Matthew Martin

**Affiliations:** 1 National Center for Computational Toxicology, Office of Research and Development, US Environmental Protection Agency, Research Triangle Park, NC 27711 USA; 2 L’Oréal Safety Research Department, 1 Avenue E. Schueller, 93600 Aulnay-Sous-Bois, France; 3 Currently at Oregon State University, Corvallis, USA; 4 Currently at Scitovation LLC, Research Triangle Park, NC USA; 5 Currently at Pfizer, Inc, Drug Safety Research and Development, 445 Eastern Point Road, MS 8274-1224, Groton, CT 06340 USA

**Keywords:** Predictive toxicity, Systemic effects, Effect levels

## Abstract

In an effort to address a major challenge in chemical safety assessment, alternative approaches for characterizing systemic effect levels, a predictive model was developed. Systemic effect levels were curated from ToxRefDB, HESS-DB and COSMOS-DB from numerous study types totaling 4379 in vivo studies for 1247 chemicals. Observed systemic effects in mammalian models are a complex function of chemical dynamics, kinetics, and inter- and intra-individual variability. To address this complex problem, systemic effect levels were modeled at the study-level by leveraging study covariates (e.g., study type, strain, administration route) in addition to multiple descriptor sets, including chemical (ToxPrint, PaDEL, and Physchem), biological (ToxCast), and kinetic descriptors. Using random forest modeling with cross-validation and external validation procedures, study-level covariates alone accounted for approximately 15% of the variance reducing the root mean squared error (RMSE) from 0.96 log_10_ to 0.85 log_10_ mg/kg/day, providing a baseline performance metric (lower expectation of model performance). A consensus model developed using a combination of study-level covariates, chemical, biological, and kinetic descriptors explained a total of 43% of the variance with an RMSE of 0.69 log_10_ mg/kg/day. A benchmark model (upper expectation of model performance) was also developed with an RMSE of 0.5 log_10_ mg/kg/day by incorporating study-level covariates and the mean effect level per chemical. To achieve a representative chemical-level prediction, the minimum study-level predicted and observed effect level per chemical were compared reducing the RMSE from 1.0 to 0.73 log_10_ mg/kg/day, equivalent to 87% of predictions falling within an order-of-magnitude of the observed value. Although biological descriptors did not improve model performance, the final model was enriched for biological descriptors that indicated xenobiotic metabolism gene expression, oxidative stress, and cytotoxicity, demonstrating the importance of accounting for kinetics and non-specific bioactivity in predicting systemic effect levels. Herein, we generated an externally predictive model of systemic effect levels for use as a safety assessment tool and have generated forward predictions for over 30,000 chemicals.

**Electronic supplementary material:**

The online version of this article (doi:10.1007/s00204-017-2067-x) contains supplementary material, which is available to authorized users.

## Introduction

The strategy for the safety assessment of cosmetics ingredients significantly changed as a result of the 7th Amendment to the Cosmetics Directive (Commission 2013). This Directive, came into full effect in 2013, banned the testing of finished products and ingredients used in cosmetics on animals; therefore, cosmetics manufacturers must now use in silico and in vitro methods to determine potential risk to humans (Commission 2013). This poses a challenge to the cosmetic industry since the ban prohibited any animal testing regardless of the availability of sufficiently predictive alternative test methods. While in vitro methods for a number of toxicity endpoints, e.g., genotoxicity (Pfuhler et al. 2014), eye irritation (McNamee et al. 2009), and skin sensitization (Johansson and Lindstedt 2014) have been refined or developed for validation as replacements for in vivo assays, alternatives to repeat-dose toxicity assays are still a big challenge due to their complexity.

In addition to the cosmetics legislation, other drivers for the development of alternative testing methods are the Registration, Evaluation, Authorization and Restriction of Chemicals (REACH) legislation in Europe and the Frank R. Lautenberg Chemical Safety for the 21st Century Act in the United States (US Safe Chemicals Act). REACH was developed in 2006 [(EC) No 1907/2006] with two main objectives: improve protection of human health and the environment from potential issues stemming from the use of chemicals and increase the competitiveness of the European chemical sector. It promotes the use of alternative test methods to identify human health and environmental hazards posed by chemicals (ECHA 2006) to reduce animal testing. REACH requires all companies manufacturing or importing chemical substances of quantities more than 1 ton per year in the European Union (EU) to be registered and with each registration, chemical safety information must be provided. The vast number of animals and time needed to accomplish this is impractical; therefore, REACH regulation challenges the chemical industries to develop rapid, relevant, cost-effective alternative methods, such as in vitro assays and computational modeling, to address human health and environmental hazards. The Frank R. Lautenberg Chemical Safety for the 21st Century Act also has provisions for requiring the EPA to take action to minimize the use of animal testing and to use computational toxicology, bioinformatics and high-throughput screening approaches where the reliability and quality of information is comparable to traditional approaches (Frank 2016). The goal is to reduce the amount of animal testing needed for each safety evaluation using these alternative methods and to encourage data sharing amongst companies and stakeholders. This will require leveraging new technologies and computational modeling approaches to increase the throughput and efficiency of safety testing while reducing or eliminating the need for animal testing (Krewski et al. 2010).

Among the research programs developing and applying in vitro and computational modeling to chemical safety, the EPA’s ToxCast project uses high-throughput screening (HTS) as a means to efficiently and economically characterize the biological activity of chemicals (Dix et al. 2007; Kavlock et al. 2012). The ToxCast project progressed in two primary phases of chemical testing, with over a thousand in vitro HTS assay endpoints collected for over one thousand unique chemicals in various biochemical or cell-based assays from different assay technologies (Kavlock et al. 2012; Kleinstreuer et al. 2014; Knudsen et al. 2009; Martin et al. 2009; Sipes et al. 2013). The EPA has also built a Toxicity Reference Database (ToxRefDB), a reference database with over 30 years of legacy animal toxicity studies containing detailed information on over 6000 in vivo animal toxicity studies on over 1100 chemicals (Judson et al. 2010; Martin et al. 2011; Reif et al. 2010; Shah et al. 2011; Sipes et al. 2011). In addition to the EPA data resources, two other open source databases are available, namely, the Hazard Evaluation Support System (HESS-DB, http://www.nite.go.jp/en/chem/qsar/hess-e.html), and COSMOS (http://www.cosmostox.eu/what/COSMOSdb/) databases. The HESS-DB was developed to support repeat-dose toxicity assessment and aid in read across and other category approaches. The COSMOS database is a legacy of the SEURAT-1 project, a European research initiative with the long-term goal of achieving “Safety Evaluation Ultimately Replacing Animal Testing”. It contains regulatory submission and open literature data from over 12,000 toxicity studies spanning 27 endpoints with detailed protocols for ~ 1600 chemicals, including cosmetics ingredients, linking chemical structure to repeat-dose toxicity data (for a subset of the substances).

The building of a predictive model of in vivo effect levels for repeat-dose systemic toxicity is a complex process due, in part, to varying experimental design and endpoint inclusion. There have been many iterations of quantitative regression models attempted and evaluated (Hisaki et al. 2015; Mumtaz et al. 1995; Pizzo and Benfenati 2016; Rupp et al. 2010; Toropova et al. 2015; Veselinovic et al. 2016), including a public and crowd-sourced challenge (USEPA 2013). These efforts demonstrated the limited ability to model systemic toxicity; a heterogeneous and variable endpoint with only a small fraction of the overall variability being explained by the model. To expand on previous work, we developed a predictive regression model of systemic effect levels using study-level covariates (e.g., species, strain, dose-spacing and administration method) in addition to chemical-level descriptors to improve the handling of study-wise sources of variability. The chemical-level descriptors comprised modeled physical–chemical properties (Mansouri et al. 2016), calculated properties, chemotypes (Ashby and Tennant 1988; Kroes et al. 2004), ToxCast bioactivity profiles and kinetic parameters. The goal of this study was to provide chemical safety decision-makers with practical prediction outputs with quantified uncertainty. Additionally, we characterized performance bounds for modeling quantitative toxicity endpoints from animal studies, i.e., the amount of variability coming from the animal study data, as their comparison with alternative approaches will depend on understanding the variability and uncertainty.

## Materials and methods

### Data sources and integration

Publicly available data sources were collected, filtered, and integrated for developing a predictive model of study-level systemic toxicity effect level. The primary linkage between all data sources was the generic substance identification (gsid; 1:1 with CAS registry number) from DSSTox (http://www.epa.gov/ncct/dsstox). All combined data and scripts are publicly available at goo.gl/R5XmxQ.

### In vivo systemic effect level and study data

Study-level systemic effect levels were collected from three resources: ToxRefDB (Martin et al. 2009), HESS-DB (http://www.nite.go.jp/en/chem/qsar/hess-e.html), and COSMOS (http://www.cosmostox.eu/what/COSMOSdb/). For all database sources, studies were filtered based on common “study inclusion criteria”: (1) oral dose administration (i.e., food, water, gavage, and capsule administration methods); (2) more than one dose level; (3) basic adherence to test guideline with acceptable study quality; and (4) testing and observation of systemic effects. Systemic effects, for the purposes of this study, were defined as in-life observation or pathological finding (i.e., clinical, macroscopic, and microscopic pathology) in repeat-dose exposed first generation adult animals. Where possible, neurotoxicity findings were excluded including cholinesterase inhibition and neurobehavioral findings. Integration of these data sources was achieved by retrieving or calculating the study-level systemic effect levels along with the corresponding effect level type and qualifier. The available effect level types consisted of lowest effect level (LEL), lowest observed adverse effect level (LOAEL), and no effect level (NEL). LEL were systematically calculated as the lowest dose at which a systemic effect was observed, whereas LOAEL were retrieved from reviewed documents whereby adversity and a specific effect level were determined for systemic toxicity. NEL were set to the lowest dose tested when no treatment-related systemic toxicities were observed. All effect levels were in units of mg/kg/day or were converted from ppm to mg/kg/day using EPA standard conversions based on assumed food and water consumption. Effect levels were then log10-transformed for all subsequent evaluation and modeling. Additional study-level covariates were extracted or calculated from the source databases, including the number of dose levels, dose spacing, test substance purity, study year, the type of effect level (i.e., NEL, LEL, LOAEL), and effect level qualifier. Effect level qualifiers were assigned when the NEL was assigned to the highest dose tested or when the LOAEL or LEL was assigned to the lowest dose tested. The effect level qualifier for LOAEL and LEL was either “less than or equal to” the lowest tested dose or “equal to” based on the dose level in which the effect level was established. Whereas the NEL was only used when no effects were observed in the study and thus given the effect level qualifier of “greater than or equal to” the highest tested dose. In addition to the effect level qualifier, the following study covariates were collected for each study across all databases: study type, species, strain, administration method, dose spacing, and number of dose groups. A mean effect level for each chemical (mean effect level) was also calculated and used to bound the predictivity of the model as described below.

#### Toxicity reference database (ToxRefDB)

ToxRefDB includes study data for over 1100 chemicals evaluated in more than 6000 animal studies (Martin et al. 2009). Systemic effect levels were queried from ToxRefDB in the form of LEL or LOAEL. LOAEL values were determined for a subset of studies, primarily registrant-submitted studies of pesticide-active ingredients that were reviewed by EPA. Subsequent to applying study inclusion criteria described above, 3752 studies across 836 chemicals were included in the present study.

#### Hazard evaluation support system database (HESS-DB)

HESS-DB was developed to support repeat-dose toxicity assessment and was coupled with other knowledge bases to aid in read-across and other category approaches (http://www.nite.go.jp/en/chem/qsar/hess-e.html; Sakuratani et al. 2013). Portions of HESS-DB have been included in the OECD toolbox. HESS-DB houses detailed repeat-dose toxicity study data, including hundreds of otherwise unpublished Japanese governmental studies performed as 28-day repeat-dose rat studies. Subsequent to applying study inclusion criteria described above, 432 studies across 411 chemicals HESS-DB were included in the present study.

#### COSmetics to optimize safety database (COSMOS-DB)

COSMOS was one of seven projects forming the SEURAT-1 cluster (Gocht et al. 2015). As part of the COSMOS project, the COSMOS relational toxicity database was developed to store regulatory submission and open literature repeat-dose study findings (http://www.cosmostox.eu/what/databases/). Version 1 of the database is publicly available at http://cosmosdb.cosmostox.eu/. COSMOS database stores over 12,000 toxicity studies spanning 27 endpoints including subchronic and chronic toxicity across approximately 1600 chemicals. Subsequent to applying study inclusion criteria described above, 195 studies across 141 chemicals were included in the present study.

### Chemical-level data

#### Physicochemical property (physchem) descriptor set

A set of physicochemical and environmental fate properties, LogP (logp), fish bioconcentration factor (bcf), water solubility (watersol), Henry’s Law constant (Henry), biodegradability (bio), fish biotransformation (biotrns), gas-phase reaction rate (aop), melting point (mp), boiling point (bp), carbon-normalized soil sorption (koc), octanol–air coefficient (koa), and vapor pressure (vp), were calculated using OPERA, a free and open source tool developed at NCCT (https://github.com/kmansouri/OPERA.git). The calculated properties are predictions of weighted k-nearest neighbors (kNN) QSAR models adapted from the publicly available PHYSPROP database in EPIsuite. Available at http://esc.syrres.com/interkow/EpiSuiteData_ISIS_SDF.htm, Scientific Databases available at http://www.srcinc.com/what-we-do/environmental/scientific-databases.html. Prior to modeling, the PHYSPROP database has undergone extensive curation using an automated KNIME workflow designed for the purpose of validating and correcting the chemical structures and their identifiers such as CAS, names, SMILES and MOLBlocks (Mansouri et al. 2016). The curated PHYSPROP datasets were then processed through a standardization workflow to generate the QSAR-ready structures used for modeling (Mansouri et al. 2016). The same standardization workflow was used to process the chemicals structures of this study prior to prediction resulting in a total of 12 modeled physchem descriptors.

#### PaDEL descriptor set

The curated molecular structures were used to calculate molecular descriptors using the free and open-source software PaDEL (Yap 2011). In PaDEL, only 2D descriptors were selected since 3D descriptors can affect the repeatability of the predictions due to differences in descriptor values, especially with very flexible molecules with a high number of 3D conformers. A total number of 1446 molecular descriptors were calculated including constitutional, topological, functional group counts, fragmental, atom-type, and E-state indices. Padel descriptors with constant or near constant values across the full chemical set were removed resulting in 1096 descriptors moving forward in the modeling process.

#### ToxPrint chemotype descriptor set

The curated molecular structures were imported into the ChemoTyper application (https://chemotyper.org/). ToxPrint chemotype fingerprints were assigned across three libraries: generic structural fragments, Ashby–Tennant genotoxic carcinogen rules (Ashby and Tennant 1988), and cancer threshold of toxicological concern (TTC) categories (Kroes et al. 2004). A total of 729 fragments were analyzed and for each chemical, the presence or absence of the fragment was recorded in a binary system as 1 or 0, respectively. ToxPrint descriptors with constant or near constant values across the full chemical set were removed resulting in 135 descriptors moving forward in the modeling process.

#### ToxCast bioactivity profiles

ToxCast HTS summarized activity calls (positive or negative) and potency estimates (the modeled 50% activity concentrations, or AC50 values) were compiled in a matrix format, with one row per chemical and columns containing assay endpoint data represented as the negative log10 of the modeled activity concentration at 50% (AC50) divided by one million. To ensure adequate assay and chemical coverage (i.e., a near complete matrix of data), chemicals with fewer than 800 assay endpoints tested or any assay endpoints with fewer than 500 chemicals tested were removed from the dataset. These assay and chemical coverage cutoffs generally equate to the full ToxCast Phase I and II chemical libraries that were screened in nearly all assays. The numeric cutoffs were used to allow for future updates and expansions to the model without having to explicitly mention the chemical library. Any spurious missing data was replaced with the assay median value, the most straightforward approach, due to the non-random and blocked nature of the missing values. For the remaining 1076 chemicals, a cytotoxicity potency estimate was calculated as previously described (Judson et al. 2016). In contrast to previous efforts that removed endpoint activity at or near cytotoxicity for a given chemical, we down-weighted such activity by subtracting out the cytotoxicity potency (i.e., “burst”). The down-weighted activity scores are also on the negative log10 scale, and such values of 0 indicate inactive, values ranging from > 0 to < 4 approximately indicate activity occurring at or near cytotoxicity, and values > 4 generally represent activity specific to the intended target. To further contextualize the assay results, assays were binned based on intended biological target or target family and assay modality (e.g., agonist vs antagonist) to form 62 assay groups and individual activity scores were averaged by target and modality. A full listing of assay to assay group mappings is provided as a supplemental table (supplementary data, Table 1). ToxCast descriptors with constant or near constant values across the full chemical set were removed resulting in 53 descriptors moving forward in the modeling process.

**Table 1:** Combined study counts across ToxRefDB, HESS-DB and COSMOS datasets by study type and species with unique chemical, strain group, and route of administration totals

Study type	Species	Study total	No. unique chemicals	No. unique strains	No. unique administration methods
SUB	Rat	774	599	5	4
CHR	Rat	566	492	5	5
DEV	Rat	542	467	5	4
CHR	Mouse	477	431	4	5
DEV	Rabbit	395	350	5	4
SAC	Rat	369	347	4	3
MGR	Rat	368	333	5	4
CHR	Dog	307	281	2	5
SUB	Mouse	263	235	4	4
SUB	Dog	200	186	2	4
DEV	Mouse	36	33	3	4
SAC	Mouse	33	30	3	3
SAC	Dog	31	31	1	4
MGR	Mouse	18	16	2	3

*SUB* subchronic (90 days), *CHR* chronic (1–2 years), *DEV* prenatal developmental, *MGR* multigenerational reproductive, *SAC* subacute (14–28 days)

#### High-throughput toxicokinetic (httk) data

Estimated toxicokinetic parameters along with two experimentally derived values, plasma protein binding and hepatic clearance, were combined in a simple model to produce oral equivalent dose values (Rotroff et al. 2010; Wetmore et al. 2012). Oral equivalent dose values are the amount of daily oral intake required to reach specific steady-state concentrations in the blood. These toxicokinetic models have been extended to also predict area under the curve (AUC), peak, and mean concentrations as well as volume of distribution in the blood assuming a specific dose regimen. The values for all subsequent modeling were calculated using the ‘httk’ R package under the assumptions of a single daily dose of 1 mg/kg/day for 90 days in humans (Pearce et al. 2017). For modeling purposes, 10 kinetic descriptors in total were selected, including intrinsic clearance, fraction unbound, area under curve (and log10 converted), peak (and log10 converted), mean (and log10 converted), and volume of distribution (and log10 converted).

#### Feature reduction and missing data handling

Following removal of descriptors for constant or near constant values, the initial descriptor set totaled 1306 descriptors. To reduce the number of descriptors prior to model development, descriptors that were highly correlated were removed using the findCorrelation function in the “caret” R package (Kuhn 2008). A correlation cutoff of 0.9 was used to identify and subsequently remove descriptors across all input descriptor sets, including physical–chemical properties (physchem), PaDEL, ToxPrint, ToxCast, and httk. The initial set of 1306 descriptors was reduced to 740 descriptors by removing 566 highly correlated descriptors. Principal component analysis was performed, using “prcomp” in R with centered and scale data, on the remaining 740 descriptors solely to characterize the remaining descriptor redundancy showing that 25% of the variance is explained in the first 4 components, 50% variance in the first 17 components, and 75% in the first 79 components. Of the remaining 740 descriptors, all missing values were replaced by the descriptor median value, the most straightforward approach, due to the non-random and blocked nature of the missing values.

### Predictive model development and evaluation

**Fig. 1:**
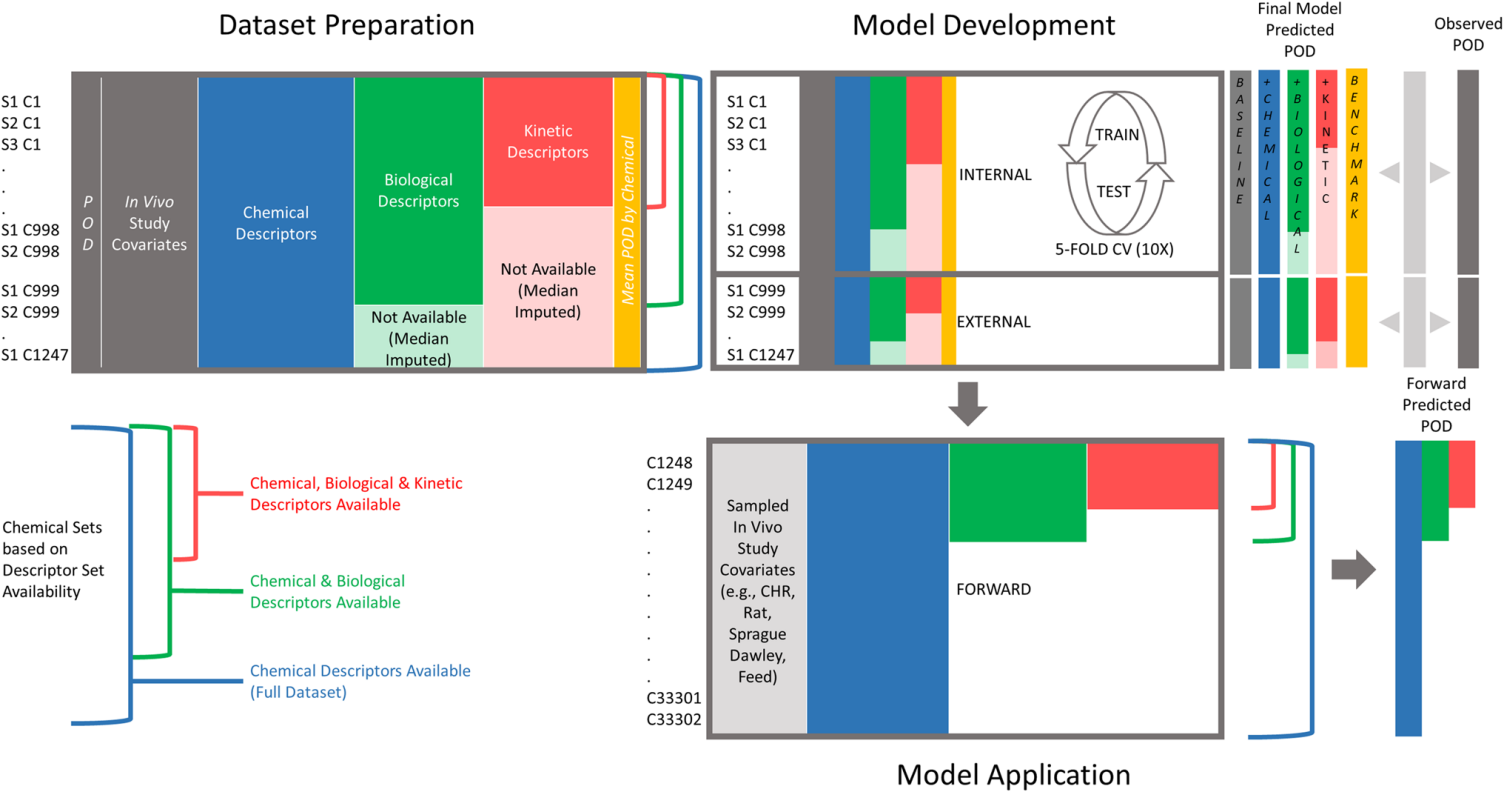
Schematic of the data preparation, model development, and model application workflow. “S” represents the study number per chemical and “C” represents the chemical index (for illustration purposes only)

A schematic of the model development process as seen in Fig. 1.

Study-level, effect level and associated study design parameters were combined by direct linkage to the gsid with the chemical-level descriptors to produce the complete dataset for modeling. All multivariate models were developed using the randomForest package in R. Additional machine learning methods were applied to the dataset and are not presented here as the random forest models were comparable or outperformed other methods in terms of reduced mean squared error (MSE); required less tuning to prevent overfitting, permitted mixed-type data; did not require data scaling; and, provided clear indications of variable importance as indicated by mean decrease in node impurity (Liaw and Wiener 2002). Throughout the model development process, two statistical methods were used to compare and evaluate model performance. Root mean squared error (RMSE) and percent variance explained (pseudo R-squared or *R* ^2^ for ease of reporting). Both statistics rely on MSE which is calculated as the mean of the squared difference of the predicted value minus the true value. RMSE is the square root of MSE while *R* ^2^ is one minus MSE divided by the variance of the observed values. Models were developed using different descriptor and chemical sets (Fig. 1). The three chemical sets stratified the study- and chemical-level data based on having only chemical descriptors, chemical and biological descriptors, and chemical, biological and kinetic descriptors; equating to sequentially smaller datasets based on data availability. Across the three chemicals sets, models were also developed using five sequentially added descriptor sets: study-level covariates only, chemical descriptors, biological descriptors, kinetic descriptors, and finally adding the mean effect level per chemical.

#### Random forest (RF) models

The complete dataset was available for modeling, which includes the feature reduced chemical-level descriptors with median imputed replacement of missing values mapped to each study effect level and their associated study-level covariates. RF, as implemented in randomForest for regression in R (Liaw and Wiener 2002), bootstraps the data, creating ‘in-bag’ and ‘out-of-bag’ sets for each tree. However, the multi-level nature of the data (i.e., study-level vs. chemical-level) presented a challenge as the inherent inter-class correlation can introduce bias and overfitting when evaluating the “out-of-bag” performance of each constructed tree, but has been shown to be addressable (Karpievitch et al. 2009). For example, if bootstrapping is performed, then training a model on study1–chemical1 and testing the performance of the model on study2–chemical1 would lead to a higher and potentially misleading assessment of the performance. To overcome this potential bias, all model development steps, including cross-validation and external validation, was performed with chemical-level splitting. Specifically, the complete dataset was split into an internal training set and an external validation set with an 80/20 split using an adaptation of the Venetian blinds technique (Consonni et al. 2009) where by the dataset was ordered by the mean effect level for each chemical (mean effect level) and every fifth chemical was selected for the external dataset. Training and testing, using the internal dataset only, was performed using randomized fivefold cross-validation and repeated five times (i.e., 5× bootstrapping). Additionally, each model set was developed against the full dataset as well as subsets of chemicals based on data availability. Performance was measured by the internal test set RMSE and *R* ^2^. RF models were developed with sequentially adding descriptor sets in following order: in vivo study covariates only (baseline model), chemical descriptors (physchem, PaDEL, ToxPrint), biological descriptors (i.e., ToxCast bioactivity), kinetic parameters (i.e., httk), and a benchmark model. The benchmark model used the mean effect level for each chemical across its respective study set (mean effect level) in addition to in vivo study covariates in developing the RF models. The baseline and benchmark models provide an estimation of the lower and upper performance bounds, respectively, to aid in assessing the quality of the primary, descriptor-based models. Specifically, the benchmark model is assuming that one would know the ‘true’ effect level for a chemical prior to developing the model and that any remaining error not explained at the study-level using the study covariates is unexplained variability. Three different chemical sets were also used in the model development process to account for the varying overlap of a specific chemical with a set of descriptors and to evaluate the relative impact of smaller datasets. The randomForest parameter, mtry, was set to one-third the number of input variables (rounded down where fractional) with the number of trees set to 250. Bias correction was performed using the linear regression coefficients (i.e., slope and intercept) of the training prediction versus observed values to adjust the test prediction values.

Final study-level RF regression models were developed using the entire internal dataset with the number of trees set to a high number (i.e., 2500 trees) to achieve more stable variable importance estimates. Variable importance was measured by the mean decrease in node impurity as indicated by the residual sum of squares (Liaw and Wiener 2002). Bias correction was performed using the mean of linear regression coefficients (i.e., mean of slope and intercept) across the fivefold CV and 5× bootstrap procedures. Final models were then applied to the external validation set to evaluate model performance of the models and RMSE and *R* ^2^ were reported. Model performance was then conducted at the chemical-level by calculating the minimum observed and predicted effect level per external test set chemical.

Forward predictions were made for 33,302 chemicals where, at minimum, the full set of chemical descriptors (i.e., physchem, PaDel and ToxPrint) were generated. Of the 33,302 chemicals with the full set of chemical descriptors, 295 chemicals were also tested in ToxCast across enough assays to derive the biological descriptors used in the current study. Of the 295 chemicals with chemical and biological descriptors, kinetic descriptors were available and modeled for 90 chemicals. Using a sampling (*N* = 5) of all observed combinations of in vivo covariates where a LOAEL was established, multiple study-level predictions were made for each chemical and the minimum, mean and standard deviation of the predicted effect levels across the sampled mock study covariates was used to represent the chemical-level predicted effect level. Uncertainty estimates were globally applied as plus or minus the model’s external test set RMSE.

## Results

### Study and chemical summary statistics

The integration of ToxRefDB, HESS-DB and COSMOS resulted in a dataset of 4379 studies across 1247 chemicals. ToxRefDB comprised the largest study set following quality and applicability filters with 3752 studies (836 chemicals). HESS-DB and COSMOS contained 433 and 195 studies (411 and 141 chemicals), respectively. Effect level data relevant to systemic toxicity were extracted from a diverse set of studies spanning multiple study types, species, strains, and routes of administration (Table 1).

**Fig. 2:**
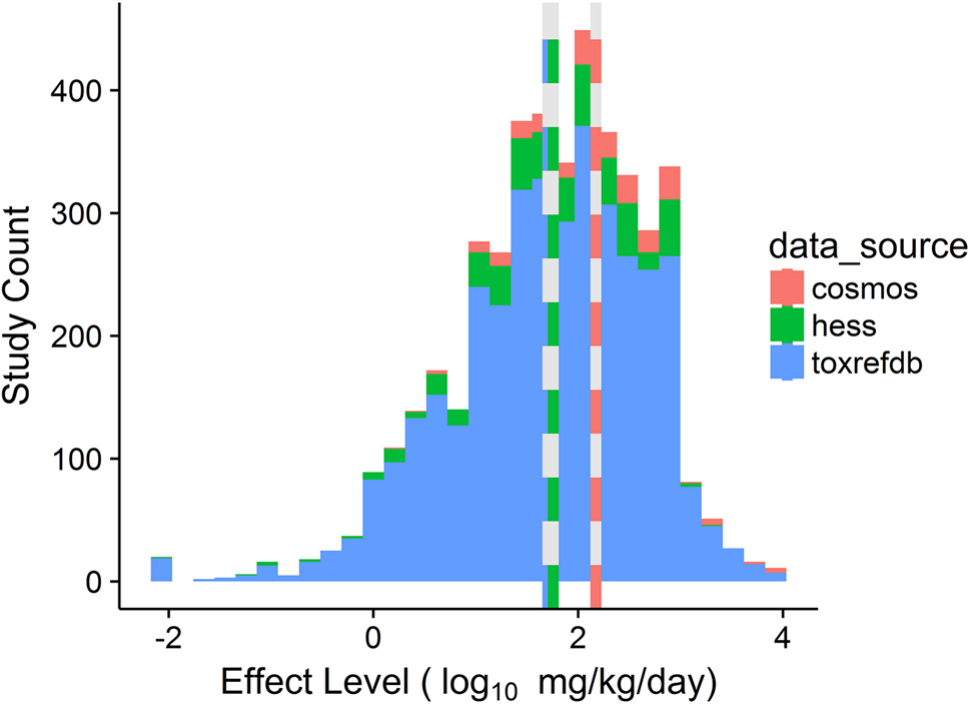
Histogram of study-level effect level (log_10_ mg/kg/day) across 4379 animal toxicity studies. The overall effect level distribution constituted a mean of 1.7 log_10_ mg/kg/day (~ 50 mg/kg/day) with a standard deviation of 0.94 log_10_ mg/kg/day. ToxRefDB and HESS-DB had comparable mean effect level of 1.7 (blue dashed line) and 1.8 (green dashed line), respectively, whereas COSMOS had a mean effect level of 2.2 log_10_ mg/kg/day (salmon dashed line)

The effect level distribution for all 4379 studies had a mean of 1.7 log_10_ mg/kg/day (~ 50 mg/kg/day) with a standard deviation of 0.94 log_10_ mg/kg/day (Fig. 2). The effect levels were truncated between −2 and 4 (0.01–10,000 mg/kg/day) to prevent disproportionate influence of extreme values with 95% of the values falling between −0.3 and 3.2 log_10_ mg/kg/day (~ 0.5 and 1500 mg/kg/day). The mean of the effect level distribution decreased significantly (*p* value ⋘ 0.01) from COSMOS (2.2 log_10_ mg/kg/day or ~ 158 mg/kg/day) as compared to HESS-DB (1.8 log_10_ mg/kg/day or ~ 55 mg/kg/day) and ToxRefDB (1.7 log_10_ mg/kg/day or 50 mg/kg/day); this observation was likely driven by differences in the chemical use types between these databases, with enrichment of cosmetic ingredients in COSMOS as compared to industrial chemicals and pesticides in HESS-DB and ToxRefDB, respectively.

**Fig. 3:**
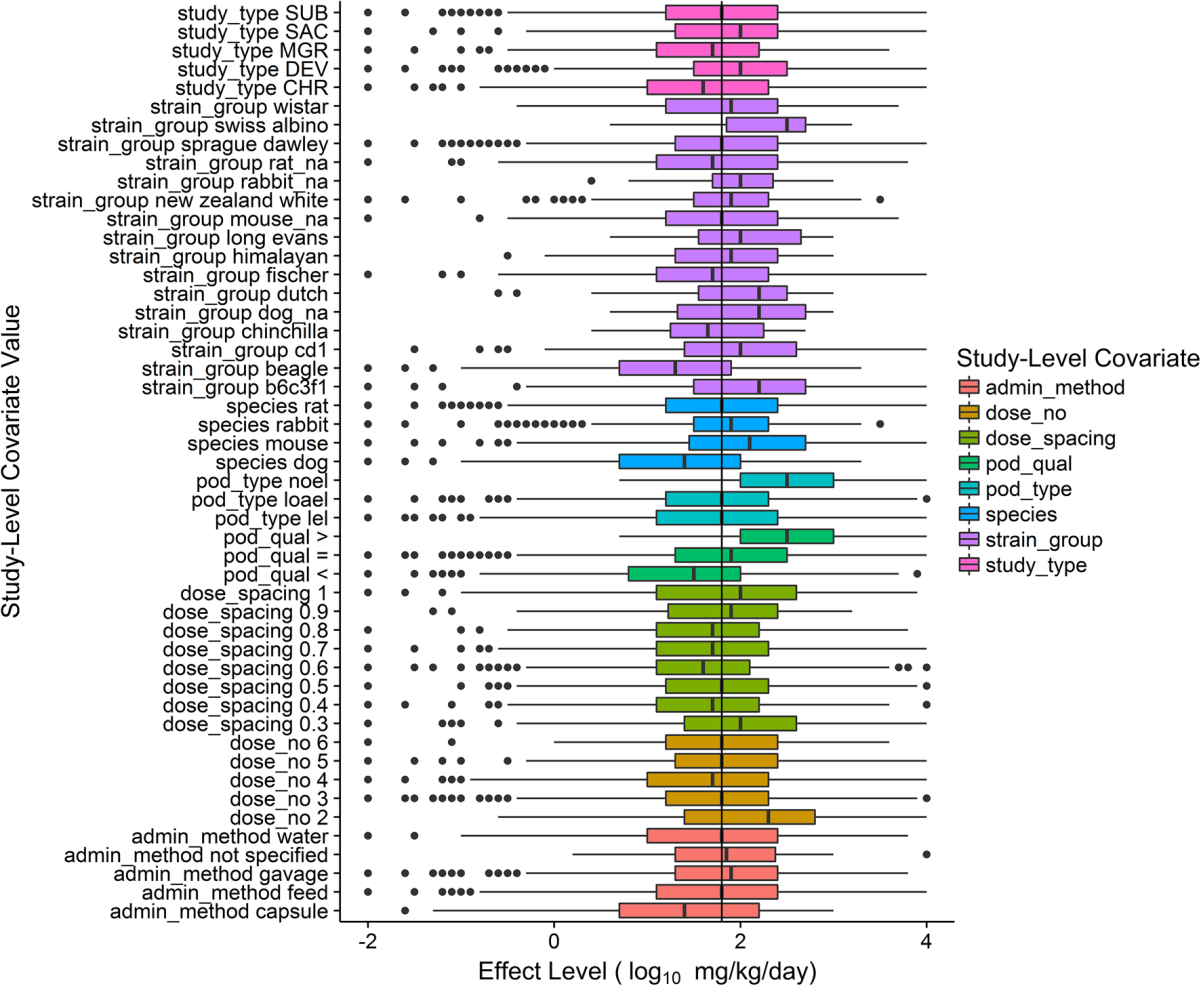
Boxplot of study-level effect level (log_10_ mg/kg/day) stratified by individual covariate values. Continuous values were binned for presentation purposes (e.g., dose spacing). The upper and lower hinges (i.e., box) correspond to the first and third quartiles, while the upper and lower whiskers correspond to the highest and lowest values, respectively, within 1.5 of the inter-quartile range. Data beyond the whiskers are shown as dots. The over effect level median of 1.8 log_10_ mg/kg/day is shown with the black vertical line

The distribution of effect levels stratified by the various study-level covariates illustrate the influence those parameters have on the overall effect level distribution (Fig. 3). The first and third quartiles (colored segments of boxplots) include the overall median effect level, 1.8 log_10_ mg/kg/day, with a few exceptions. Notably, the NEL effect levels have substantially higher values with a median of 3, a log_10_ dose equivalent to 1000 mg/kg/day, or the top allowable dose for most guideline toxicity studies. The individual covariate distributions demonstrate the average impacts of various study-level parameters and provide support for not applying standard safety (i.e., conversion) factors based simply on study type, duration and species.

The chemical-level dataset (chemical, biological and kinetic descriptor sets) was integrated via chemical to the study-level dataset. However, biological and kinetic descriptor sets were only available for subsets of chemicals and, hence, studies (Table 2). The respective study and chemical counts as additional descriptor sets were added to illustrate the tradeoff in expanding the diversity of descriptors at the expense of study and chemical coverage.

**Table 2:** Study and chemical counts based on availability of descriptor sets across the 4379 studies and 1247 chemicals with internal and external dataset counts provided

Study and chemical sets based on descriptor set availability	Study count (internal/external)	Chemical count (internal/external)	Descriptor count including study covariates
Chemical descriptors available	4379 (3476/903)	1247 (998/249)	699
Chemical and biological descriptors available	3106 (2427/679)	603 (468/135)	742
Chemical, biological, and kinetic descriptors available	2189 (1688/501)	391 (304/87)	748

With the addition of each subsequent descriptor set the total number of descriptors increased while study and chemical counts decreased

### Systemic effect level models

#### Internal training and testing across all descriptor sets

**Fig. 4:**
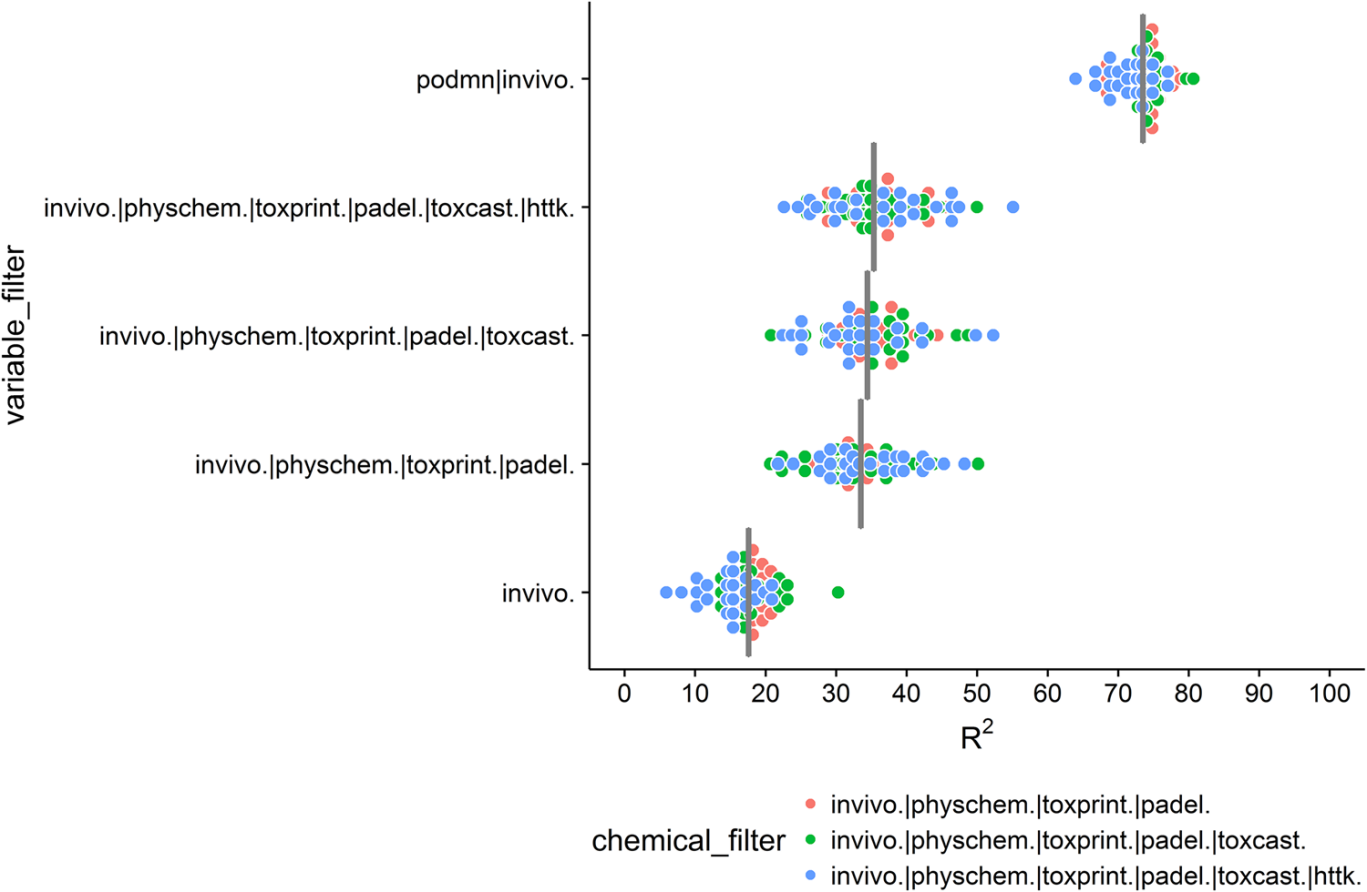
Dotplot of variance explained (*R* ^2^) of cross-validation test sets for each bootstrap (*n* = 5) across five model sets including baseline and benchmark models. The five model sets were also run with varying chemical sets based on availability of biological and kinetic descriptors. The gray crossbar has been placed at the median *R* ^2^ for each of the five model sets

Cross-validation models were developed containing study-level covariates with sequentially added descriptor sets for the three chemical sets (Fig. 4). Performance was compared based on the percent variance explained (*R* ^2^) from the fivefold cross-validation test sets for each bootstrapped dataset (*n* = 5) illustrating the relative stability of the models developed using different chemical and descriptor sets. Each model expanded the descriptor set beyond the original eight study-level covariates with an additional 12 physchem, 554 PaDEL, 119 ToxPrint, 49 ToxCast, and 6 httk descriptors. The baseline model was developed using only the study-level covariates (i.e., in vivo covariates only) and resulted in a median *R* ^2^ of 18% across all cross-validated and bootstrapped datasets and chemical sets. The three primary model sets improved over the baseline performance with a median *R* ^2^ of 35% demonstrating model stability. However, the addition of biological and kinetic descriptors did not significantly improve model performance. Adding biological or kinetic descriptors alone without chemical descriptors does improve model performance over baseline (data not shown). The benchmark model was developed using study-level covariates and chemical-level effect level (i.e., mean effect level across all studies for each chemical) which accounted for 74% of the total variance. Internal model training and cross-validation detailed relative model performance and provided context for evaluating the final models.

#### Final model development

Final models were developed using the full internal data set with the external dataset of 858 studies across 240 chemicals characterizing the overall model performance and uncertainty. The final RF models were also developed using a large number of trees (ntree = 2500) for a robust evaluation of descriptor importance. Model performance (i.e., external test set *R* ^2^ and RMSE) and the top five additional descriptors with their relative rank amongst all descriptors are summarized in Table 3. Variable importance plots are also available (Supplementary Data, Fig. 1). All models have significantly increased *R* ^2^, 32–43%, as compared to the baseline performance metric (*R* ^2^ = 11–16%) established by developing a model only using in vivo covariates. The performance metrics were also highly comparable between the cross-validated training models and the final models. The collection of models did not approach the benchmark performance metric of approximately 70% variability explained. The roughly 30% gap in explained variability between the primary models and the benchmark model was likely due, in part, to unquantified and unaccounted variability in the observed effect levels (e.g., vehicle control substance of each study, animal handling procedures, data reporting protocols). Additionally, uncertainty in the input descriptors (e.g., noise/error in the predicted physical chemical properties and ToxCast results) also contributed to the performance gap. Nonetheless, chemically, biologically and kinetically plausible descriptors were demonstrated to be of high relative importance, e.g., logP, physico-chemical property and “burst” activity in ToxCast. Study-level covariates consistently remained, as expected, highly important in each model with dose-spacing being among the top five most important descriptors in all models, underscoring the influence of dose-spacing in effect level determinations. The chemical descriptor model highlighted descriptors recognized to be important in driving chemical distribution and uptake including bioconcentration factor (bcf) and bioavailability (bio). Additionally, the PaDEL descriptor set added autocorrelation metrics (ATSC4m and AATSC1m) and logP (ALogP). The ToxPrint descriptor set was generally less important but did reflect that organophosphate structures (bond.P.S_generic) corresponded to lower effect level, reflective of the acute toxicity of these chemicals. Even though biological and kinetic descriptors did not improve overall model performance, ToxCast descriptors provided biologically relevant descriptors; all of which were plausible determinants of systemic effects including PPARα activity, zebrafish toxicity, and cytotoxicity (i.e., ‘burst’ activity, Judson et al. 2016), and estrogen receptor activity. Additionally, ToxCast descriptors provided a metabolic context with xenobiotic metabolism induction as a highly important descriptor. The preliminary addition of kinetic (httk) descriptors for a relatively small number of chemicals (322 chemicals in the internal dataset and 74 in the external dataset) showed marginal variable importance in combination with all other input descriptors. The underlying httk data and models have and continue to be expanded, improved and evaluated for chemical-specific reliability. For the purpose of this study, only publicly available and unfiltered data was used in an attempt to allow for immediate public use as well as to maintain as much chemical overlap as possible. The relationship between modeled kinetic descriptors and effect levels requires further exploration.

**Table 3:** Final model performances (ntree = 2500) using the full chemical set with *R* ^2^ and RMSE of the external test set presented

Terminal descriptor set	Study-level model performance			Top 5 descriptors from terminal descriptor set (importance rank)	Chemical-level model performance		
	RMSE	*R* ^2^	SD		RMSE	*R* ^2^	SD
In vivo study covariates only (baseline)	0.85	16	0.93	dose_spacing (1); strain_group (2); pod_qual (3); study_type (4); dose_no (5)	0.91	13	0.98
+ chemical descriptors (physchem, PaDEL, ToxPrint)	0.7	43	0.92	AATSC0p (6); MDEO-11 (7); ATSC4 m (9); SHsOH (10); SHBd (13)	0.73	48	1
+ biological descriptors (ToxCast)	0.7	43	0.92	peroxisome_proliferator_activated_receptor_alpha (25); estrogen_receptor (48); xenobiotic_metabolism_induction (87); zebrafish_development (269); androgen_receptor (364)	0.73	48	1
+ kinetic descriptors (httk)	0.69	43	0.92	logmean (124); intcl (137); peak (213); logvdist (263); fub (295)	0.73	49	1
+ mean effect level (benchmark)	0.5	72	0.93	podmn (1)	0.4	83	0.98

The standard deviation of the observed effect levels is also shown to provide context to the RMSE values. The top five descriptors of the sequentially added descriptor sets with their variable importance rank illustrated the relative impact of the additional descriptor set. Using the minimum of the observed and predicted study effect levels per chemical, model performance was evaluated at the chemical-level

*SD* standard deviation of observed values (log_10_ mg/kg/day)

**Fig. 5:**
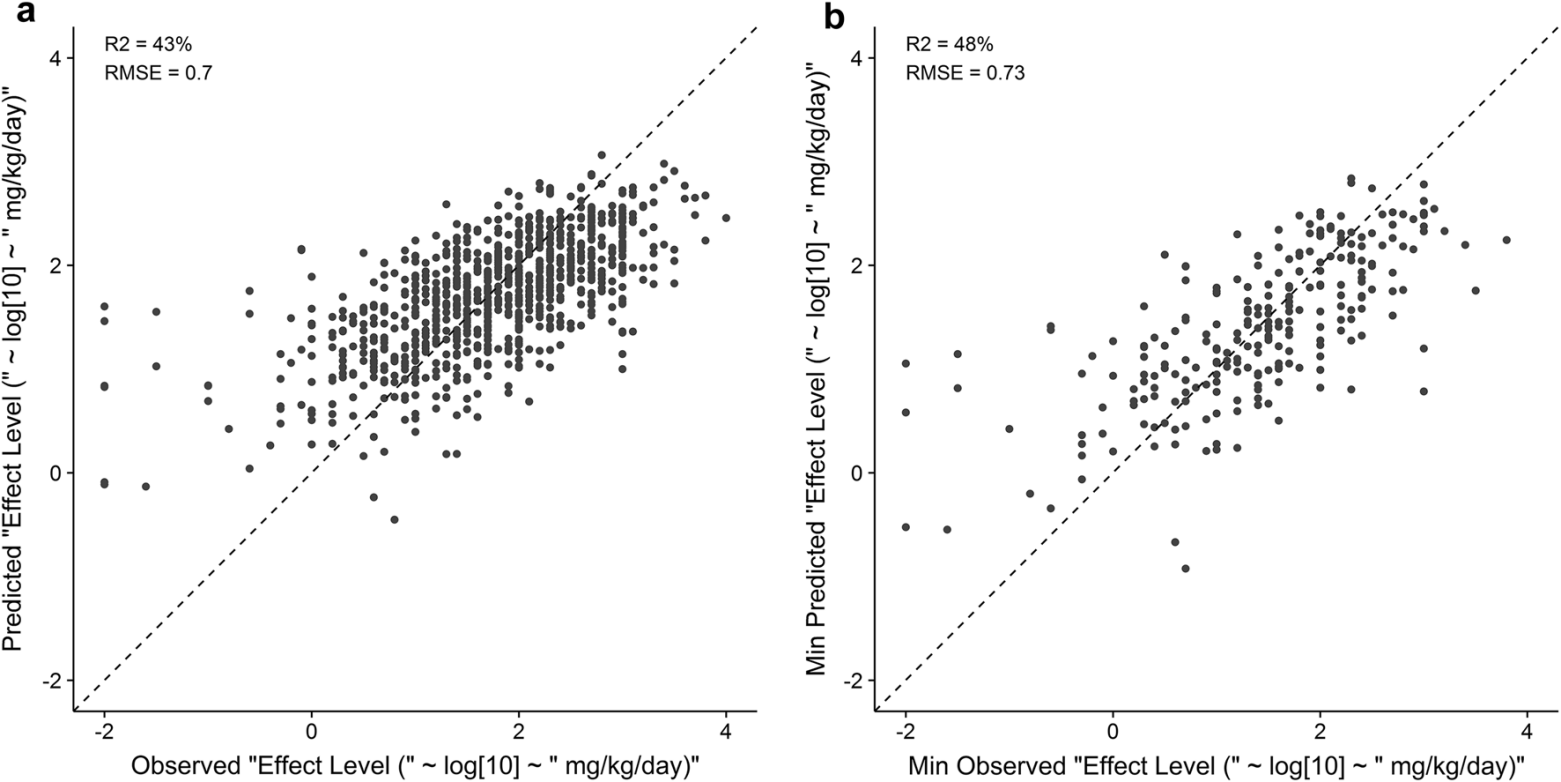
**a** Predicted vs observed study effect level (log_10_ mg/kg/day) of the full external test set (*N* = 903 studies) using study-level covariates with chemical, biological and kinetic descriptors resulted in an *R* ^2^ of 43% and an RMSE of 0.7 log_10_ mg/kg/day. **b** Using the minimum predicted and observed effect level per chemical in the external test set (*N* = 249 chemicals), chemical effect level predictions were made and resulted in an *R* ^2^ of 48% and an RMSE of 0.73 log_10_ mg/kg/day

Model predictions across the full external test set of 903 studies using the full complement of chemical, biological, and kinetic descriptors resulted in an *R* ^2^ of 43% and an RMSE of 0.69 log_10_ mg/kg/day (Fig. 5a). Of the 903 effect level predictions, 788 predicted effect level (87%) were within an order of magnitude of the observed effect level. Practically, the utility of the model predictions would be for decisions made at the chemical-level. Comparing the minimum observed effect level and minimum predicted effect level for the 249 chemicals in the external test set resulted in an *R* ^2^ of 48% and an RMSE of 0.73 log_10_ mg/kg/day demonstrating the practical utility of the model outputs for chemical-specific predictions (Fig. 5b). Similar to the study-level predictions, approximately 87%, or 216 out of the 249, external test set chemicals, were within an order of magnitude of the minimum observed effect level. Only 6 out of 249 chemicals had predictions greater than two orders of magnitude from the minimum observed effect level. It should be noted that 5 out of those 6 chemicals were under-predicted demonstrating the challenge of detecting and quantifying extreme values.

#### Forward predictions

Forward predictions were made across the applicable set of descriptors and chemicals (i.e., the subset of chemicals that have chemical, biological and/or kinetic descriptors). For each chemical, a random sampling of five mock studies across all observed combinations of in vivo covariates with an established LOAEL were generated and combined with the chemical-level descriptors to make study-level predictions. The sampling of observed in vivo covariates represented the range of possible study conditions for which any future chemical could undergo. Models developed using the sample observed in vivo covariates and chemical descriptors only generated predictions for 31,302 chemicals. Therefore, for each of the 31,302 forward predictions chemicals there were a total of 156,510 total study-level effect level predictions to provide a distribution of predicted values across a diverse set of study conditions. The minimum predicted effect level across the sampled study covariates was selected to represent the chemical-level effect level and is provided as a supplemental file (supplementary data, Table 2). Additionally, the mean and standard deviation of the study-level predicted effect level is provided to illustrate the relative impact of study conditions on the predictions. Future predictions were also made using chemical and biological descriptors on 295 chemicals and on 90 chemicals with kinetic descriptors.

## Discussion

At face value, this work demonstrated the marginal predictivity of regression-based, random forest models of systemic effect levels using a collection of descriptors. However, this work also demonstrated the importance of accounting for study-level covariates within the modeling process and estimating performance expectation bounds to assess model utility. The modeled dataset comprised of 4379 studies 1247 chemicals curated from three different database resources: ToxRefDB, HESS-DB, and COSMOS. Effect levels (log_10_ mg/kg/day) were randomly divided into an internal (3476 studies of 998 chemicals) and external dataset (903 studies of 249 chemicals) with no single chemical in both the internal and external set. The databases cover a diverse set of chemicals spanning pesticides, industrial chemicals, cosmetics, and pharmaceuticals and are generally underrepresented by highly toxic compounds (e.g., dioxin) to avoid over-weighting of extreme values. Chemical, biological, and kinetic descriptors were applied if available. A novel step in this analysis was the use of study-level covariates in the model development process. The internal dataset was used to develop a suite of models with fivefold cross-validation and 5X bootstrapping to test overall model performance and stability. The full internal dataset was then used for final model development. The results were very similar to the cross-validation models demonstrating the robustness of the modeling approach and that overfitting was unlikely to have a significant impact on future performance. The baseline and benchmark models provided lower and upper performance bounds with RMSE estimates of 0.85 and 0.5 log_10_ mg/kg/day, respectively, equating to approximately 15 and 70% variance explained (*R* ^2^). The final consensus model, including chemical, biological, and kinetic descriptors, had an RMSE of 0.69 log_10_ mg/kg/day explaining 43% of the study-level effect level variance.

Performance metrics of regression models are often reported as RMSE and *R* ^2^. However, these metrics do not provide the full context of model performance, as they do not take into account underlying expectations and upper performance bounds for how good a model could be given the underlying data. Effect levels, in particular, are associated with a large amount of variability due to study design and interpretation of the observations that form the basis for assigned NOAEL, LOAEL, NEL, or LEL values (Leisenring and Ryan 1992). Effect levels extracted from various data sources also carry with them a level of ‘unexplainable’ variability, as the source of the variability may not be captured in the primary reports, study summaries, or a computable format in database. For example, animal handling technique and expert contributions to LOAEL selection contribute to systematic study error or bias not generally captured. Therefore, the expectation to approach 100% explained variance (i.e., RMSE approaching 0 or *R* ^2^ approaching 100%) is unreasonable and would be a clear sign of overfitting. Baseline and benchmark RF models were developed incorporating the study-level covariates (e.g., study type, species, strain) with and without the chemical-level effect levels (i.e., mean effect level across all studies for each chemical). Even though regulatory applications would not utilize the chemical-level mean effect level, the mean effect level is used as an anchoring point to assess the variability across study type, species, etc., from chemical-to-chemical. The baseline RF model showed that approximately 15% of the variance was explained by study-level covariates and adding chemical, biological, and kinetic descriptors resulted in increased *R* ^2^ and decreased RMSE. The benchmark RF model explained approximately 70% of the variability, providing an estimated upper bound within which even a model with perfect input parameters (i.e., zero uncertainty in the input data fully explaining all dynamic and kinetic factors of effect level determinations) would not be expected to exceed. Baseline and benchmark performance metrics established by these methods serve as guideposts for contextualizing the final models. However, the benchmark estimate is likely overoptimistic as the mean effect level was calculated using the observed, not true, effect level. A more detailed and thorough examination of sources of effect level variability beyond the baseline and benchmark models presented herein is needed and underway.

Initially, we and many other groups modeled effect levels from each study at the chemical-level (i.e., prediction of minimum effect level across all studies by chemical) with limited success (Novotarskyi et al. 2016; USEPA 2013). The effect levels used for the initial modeling effort, derived from many different study types and spanning many repeat-dose systemic effect observations, constituted a heterogeneous endpoint for prediction. The current work attempted to address the heterogeneity in the source data by modeling effect levels at the study-level as opposed to the chemical-level. Study-level effect levels and associated study-specific covariates were combined with chemical-level descriptors for the development of a set of predictive random forest models. This methodology enabled accounting for variability in the study type and other study-level covariates. Although not directly comparable, the chemical-level predictions from the EPA challenge (Novotarskyi et al. 2016; USEPA 2013) resulted in final models that explained roughly 30% of the variance, while the chemical-level predictions from the current model explain nearly 50% of the variance in the external validation dataset. There is a long history of attempting to develop QSAR and other predictive models of NOAEL, LOAEL, LD_50_, and other effect levels (Hisaki et al. 2015; Mumtaz et al. 1995; Pizzo and Benfenati 2016; Rupp et al. 2010; Toropov et al. 2015; Veselinovic et al. 2016), and the summary performance statistics for these published models vary widely. Additionally, external evaluation of these models has often shown that the models either only apply to a very specific chemical domain or were originally reported with overoptimistic performance statistics (Pizzo and Benfenati 2016). Although attempted, direct comparisons to these previous models were not made due to a number of limiting factors, including different endpoints (e.g., NOAEL/LOAEL vs LD_50_), lack of cross- and/or external validation, availability of underlying chemical or toxicological data, and very limited chemical space). Therefore, in addition to developing the model using study-level covariate information, a focus of the present work was to provide performance baseline and benchmark guideposts using the data in-hand.

Similar to the findings and observations of Novotarskyi et al. (2016), adding biological descriptors, i.e., ToxCast bioactivity data, did not significantly increase model performance. There is limited, peer-reviewed guidance on the incorporation of bioactivity data in modeling. The OECD has recommended a set of five guidelines for development of QSAR models, including data used be associated with a mechanistic interpretation of a predicted endpoint (Fjodorova et al. 2008). The results of the modeling process herein may provide biological plausibility and mechanistic insight into the driving systemic effects at the effect level, as nuclear receptor activity, oxidative stress, and cytoxicity were leading predictive descriptors. Additionally, an advantage to creating ToxCast assay groups, beyond reducing descriptor space and highly correlated descriptors, is the easy translation of the model for practical applications in the future. It is not necessary to run every assay in the current ToxCast suite to generate an activity score for the model and to gain biological insight. Even with a subset of the assay data, this model can be run in conjunction with chemical descriptors to predict effect levels. Assay grouping serves to outline common biological space, so HTS assays that are different from those currently in ToxCast, but that assess the same biological space or function, could be used as descriptors for model development and prediction.

A limitation of the current model is the available bioactivity descriptors from ToxCast/Tox21 represent a finite biological space currently covered in the ToxCast/Tox21 HTS program. ToxCast and Tox21 research programs continue to expand biological and technological space, including investigation into the feasibility and utility of high throughput transcriptomics and assays with increased metabolic capacity. Additionally, the current model does not take into account kinetics for the full chemical library. To ensure the model will have a broad domain of chemical applicability, future directions will include incorporation of additional reverse toxicokinetic data (Rotroff et al. 2010; Wetmore et al. 2012) to enhance the kinetic context; transcriptomics (Paules 2014) and additional HTS assays to provide broader biological coverage; and, expansion of the chemical space included in the internal validation set.

In conclusion, a novel suite of regression models of repeat-dose systemic toxicity was developed using study-level covariates and chemical-level descriptors capable of predicting effect levels with quantified uncertainties. Forward predictions were made for over 30,000 chemicals, many of which have little to no empirical bioactivity or toxicity data. This work demonstrates it is possible to predict effect levels for additional chemicals if the appropriate chemical descriptor sets are available. Potential applications of this model include use in weight-of-evidence evaluations for chemicals that are barred from use in animal testing, such as cosmetic ingredients being developed in Europe. Within the US, the model could be used to predict effect levels for data poor chemicals in the home or environment (e.g., contaminated sites), fill in gaps for estimating the hazard index for multiple contaminants, or in emergency situations for chemicals with limited data. Regardless, the suite of predictive systemic effect level models provides a key indication of hazard potential and dose response characterization for thousands of chemicals with limited safety-related data.

## Electronic supplementary material

Below is the link to the electronic supplementary material.

Supplementary material 1 (PDF 33 kb)

Supplementary material 2 (CSV 41 kb)

Supplementary material 3 (CSV 4854 kb)
