# Supplementary material for: Predicting in vivo effect levels for repeat-dose systemic toxicity using chemical, biological, kinetic and study covariates
Source: Arch Toxicol. 2017 Oct 27;92(2):587–600. doi: 10.1007/s00204-017-2067-x (PMC5818596; doi:10.1007/s00204-017-2067-x)
Supplement: Supplementary file 1 — Supplementary material 1 (PDF 33 kb) [file 204_2017_2067_MOESM1_ESM.pdf]

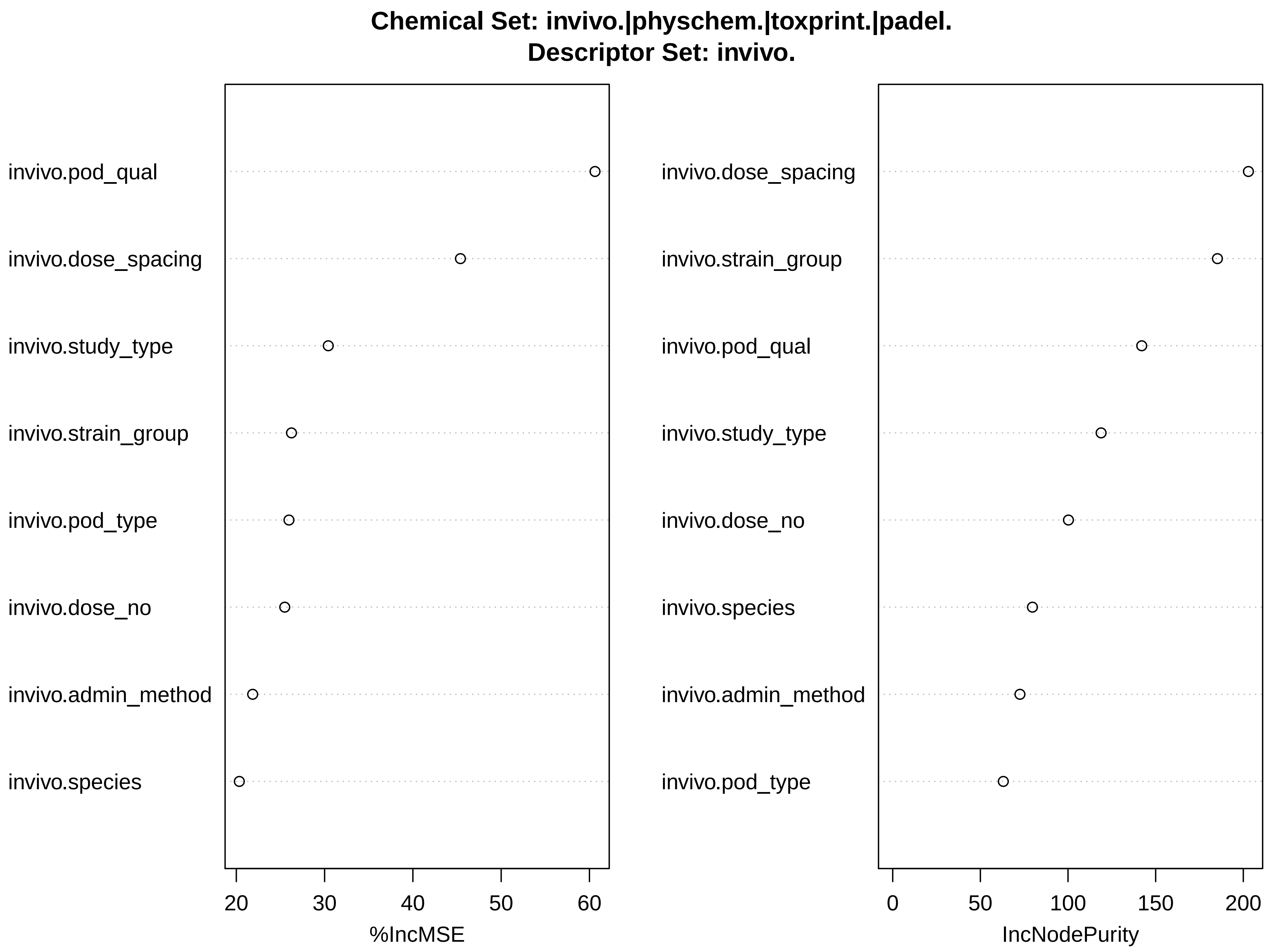
Chemical Set: invivo.|physchem.|toxprint.|padel.
Descriptor Set: invivo.
invivo.pod_qual
invivo.dose_spacing
invivo.study_type
invivo.strain_group
invivo.pod_type
invivo.dose_no
invivo.admin_method
invivo.species
20
30
40
50
60
%IncMSE
invivo.dose_spacing
invivo.strain_group
invivo.pod_qual
invivo.study_type
invivo.dose_no
invivo.species
invivo.admin_method
invivo.pod_type
0
50
100
150
200
IncNodePurity

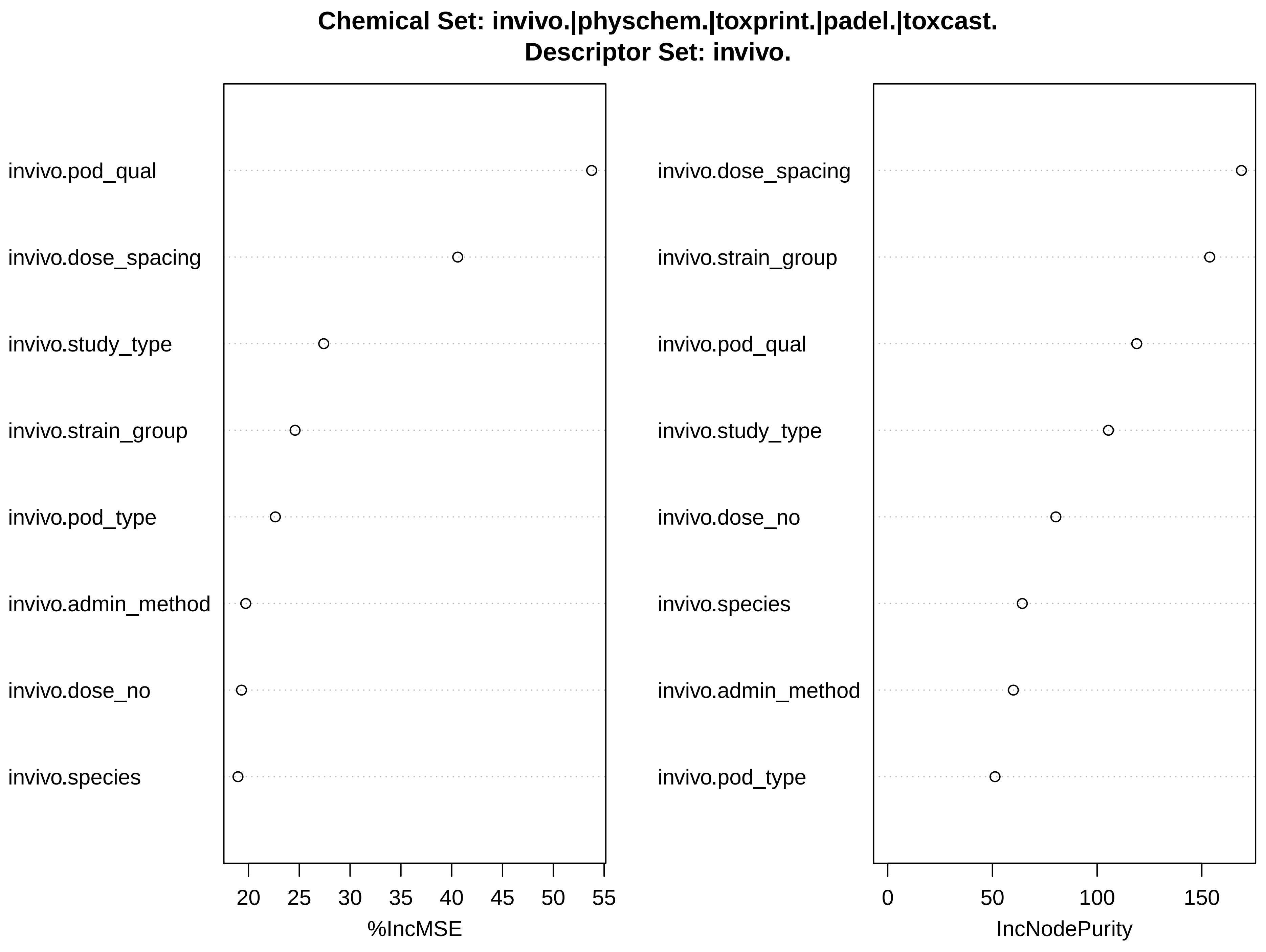

Chemical Set: invivo.|physchem.|toxprint.|padel.|toxcast.
Descriptor Set: invivo.
invivo.pod_qual
invivo.dose_spacing
invivo.study_type
invivo.strain_group
invivo.pod_type
invivo.admin_method
invivo.dose_no
invivo.species
20
25
30
35
40
45
50
55
%IncMSE
invivo.dose_spacing
invivo.strain_group
invivo.pod_qual
invivo.study_type
invivo.dose_no
invivo.species
invivo.admin_method
invivo.pod_type
0
50
100
150
IncNodePurity

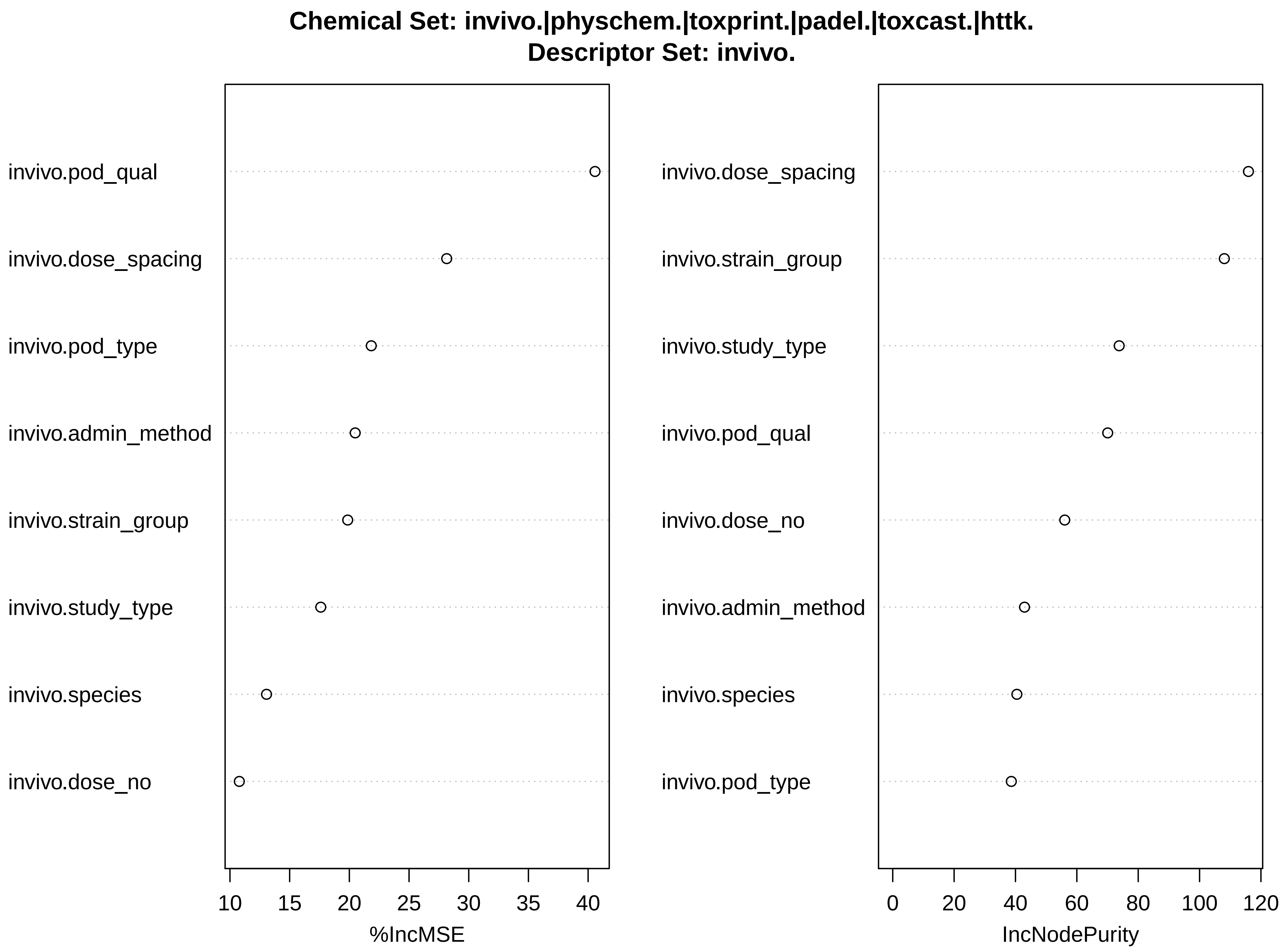
Chemical Set: invivo.|physchem.|toxprint.|padel.|toxcast.|httk.
Descriptor Set: invivo.
invivo.pod_qual
invivo.dose_spacing
invivo.pod_type
invivo.admin_method
invivo.strain_group
invivo.study_type
invivo.species
invivo.dose_no
10
15
20
25
30
35
40
%IncMSE
invivo.dose_spacing
invivo.strain_group
invivo.study_type
invivo.pod_qual
invivo.dose_no
invivo.admin_method
invivo.species
invivo.pod_type
0
20
40
60
80
100
120
IncNodePurity

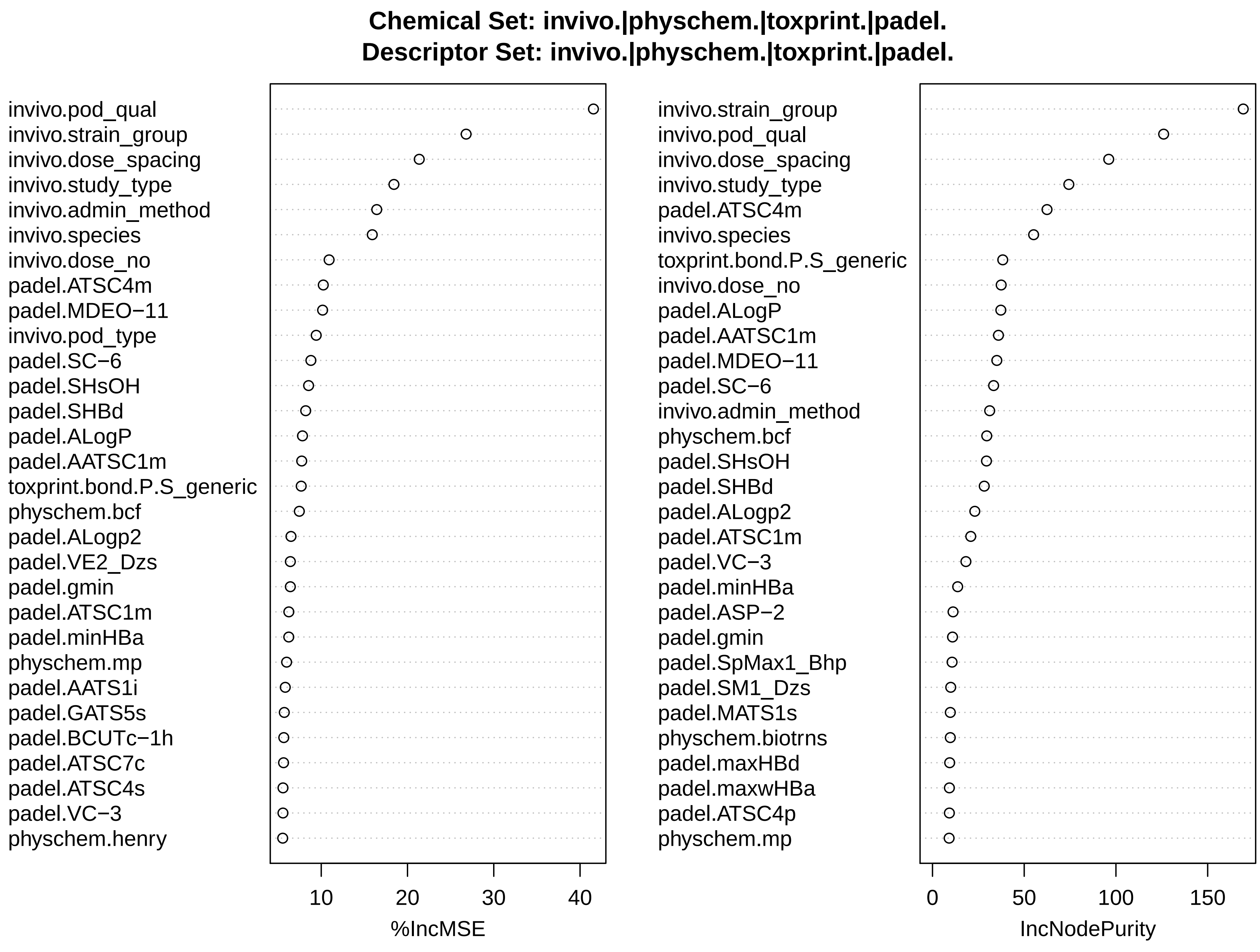

Chemical Set: invivo.|physchem.|toxprint.|padel.
Descriptor Set: invivo.|physchem.|toxprint.|padel.
invivo.pod_qual
invivo.strain_group
invivo.dose_spacing
invivo.study_type
invivo.admin_method
invivo.species
invivo.dose_no
padel.ATSC4m
padel.MDEO−11
invivo.pod_type
padel.SC−6
padel.SHsOH
padel.SHBd
padel.ALogP
padel.AATSC1m
toxprint.bond.P.S_generic
physchem.bcf
padel.ALogp2
padel.VE2_Dzs
padel.gmin
padel.ATSC1m
padel.minHBa
physchem.mp
padel.AATS1i
padel.GATS5s
padel.BCUTc−1h
padel.ATSC7c
padel.ATSC4s
padel.VC−3
physchem.henry
10
20
30
40
%IncMSE
invivo.strain_group
invivo.pod_qual
invivo.dose_spacing
invivo.study_type
padel.ATSC4m
invivo.species
toxprint.bond.P.S_generic
invivo.dose_no
padel.ALogP
padel.AATSC1m
padel.MDEO−11
padel.SC−6
invivo.admin_method
physchem.bcf
padel.SHsOH
padel.SHBd
padel.ALogp2
padel.ATSC1m
padel.VC−3
padel.minHBa
padel.ASP−2
padel.gmin
padel.SpMax1_Bhp
padel.SM1_Dzs
padel.MATS1s
physchem.biotrns
padel.maxHBd
padel.maxwHBa
padel.ATSC4p
physchem.mp
0
50
100
150
IncNodePurity

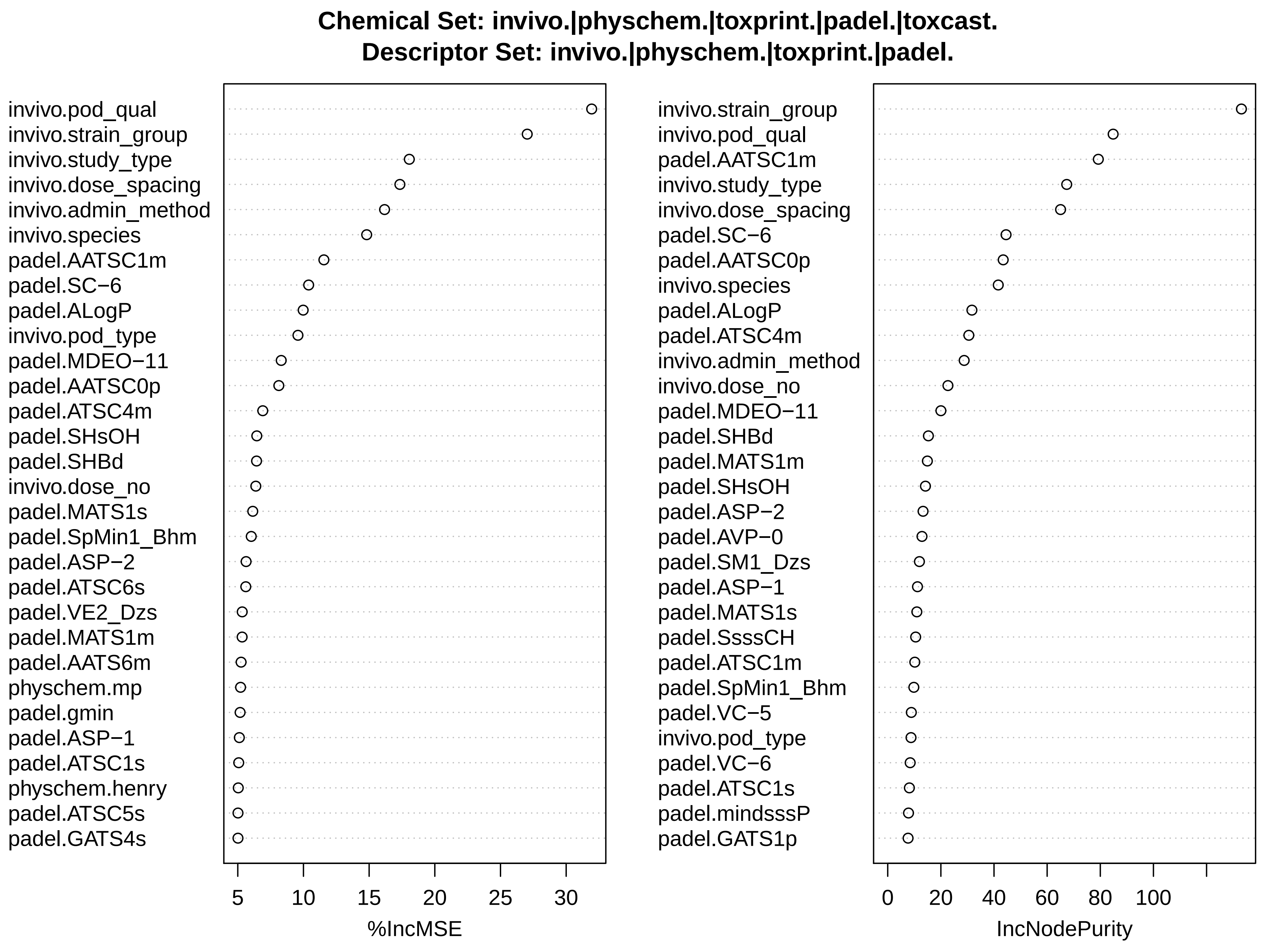
Chemical Set: invivo.|physchem.|toxprint.|padel.|toxcast.
Descriptor Set: invivo.|physchem.|toxprint.|padel.
invivo.pod_qual
invivo.strain_group
invivo.study_type
invivo.dose_spacing
invivo.admin_method
invivo.species
padel.AATSC1m
padel.SC−6
padel.ALogP
invivo.pod_type
padel.MDEO−11
padel.AATSC0p
padel.ATSC4m
padel.SHsOH
padel.SHBd
invivo.dose_no
padel.MATS1s
padel.SpMin1_Bhm
padel.ASP−2
padel.ATSC6s
padel.VE2_Dzs
padel.MATS1m
padel.AATS6m
physchem.mp
padel.gmin
padel.ASP−1
padel.ATSC1s
physchem.henry
padel.ATSC5s
padel.GATS4s
5
10
15
20
25
30
%IncMSE
invivo.strain_group
invivo.pod_qual
padel.AATSC1m
invivo.study_type
invivo.dose_spacing
padel.SC−6
padel.AATSC0p
invivo.species
padel.ALogP
padel.ATSC4m
invivo.admin_method
invivo.dose_no
padel.MDEO−11
padel.SHBd
padel.MATS1m
padel.SHsOH
padel.ASP−2
padel.AVP−0
padel.SM1_Dzs
padel.ASP−1
padel.MATS1s
padel.SsssCH
padel.ATSC1m
padel.SpMin1_Bhm
padel.VC−5
invivo.pod_type
padel.VC−6
padel.ATSC1s
padel.mindsssP
padel.GATS1p
0
20
40
60
80
100
IncNodePurity

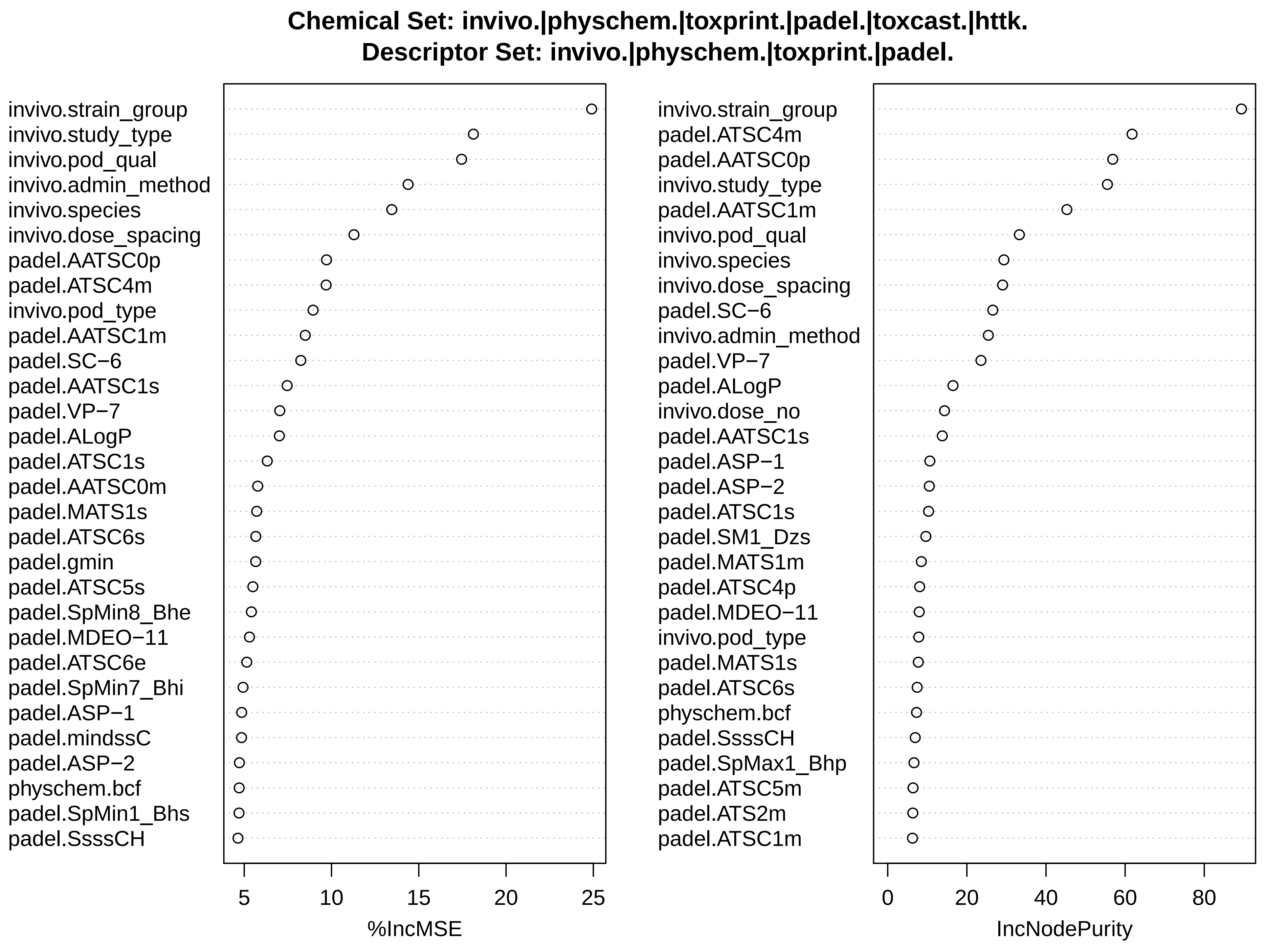
Chemical Set: invivo.|physchem.|toxprint.|padel.|toxcast.|httk.
Descriptor Set: invivo.|physchem.|toxprint.|padel.
invivo.strain_group
invivo.study_type
invivo.pod_qual
invivo.admin_method
invivo.species
invivo.dose_spacing
padel.AATSC0p
padel.ATSC4m
invivo.pod_type
padel.AATSC1m
padel.SC−6
padel.AATSC1s
padel.VP−7
padel.ALogP
padel.ATSC1s
padel.AATSC0m
padel.MATS1s
padel.ATSC6s
padel.gmin
padel.ATSC5s
padel.SpMin8_Bhe
padel.MDEO−11
padel.ATSC6e
padel.SpMin7_Bhi
padel.ASP−1
padel.mindssC
padel.ASP−2
physchem.bcf
padel.SpMin1_Bhs
padel.SsssCH
5
10
15
20
25
%IncMSE
invivo.strain_group
padel.ATSC4m
padel.AATSC0p
invivo.study_type
padel.AATSC1m
invivo.pod_qual
invivo.species
invivo.dose_spacing
padel.SC−6
invivo.admin_method
padel.VP−7
padel.ALogP
invivo.dose_no
padel.AATSC1s
padel.ASP−1
padel.ASP−2
padel.ATSC1s
padel.SM1_Dzs
padel.MATS1m
padel.ATSC4p
padel.MDEO−11
invivo.pod_type
padel.MATS1s
padel.ATSC6s
physchem.bcf
padel.SsssCH
padel.SpMax1_Bhp
padel.ATSC5m
padel.ATS2m
padel.ATSC1m
0
20
40
60
80
IncNodePurity

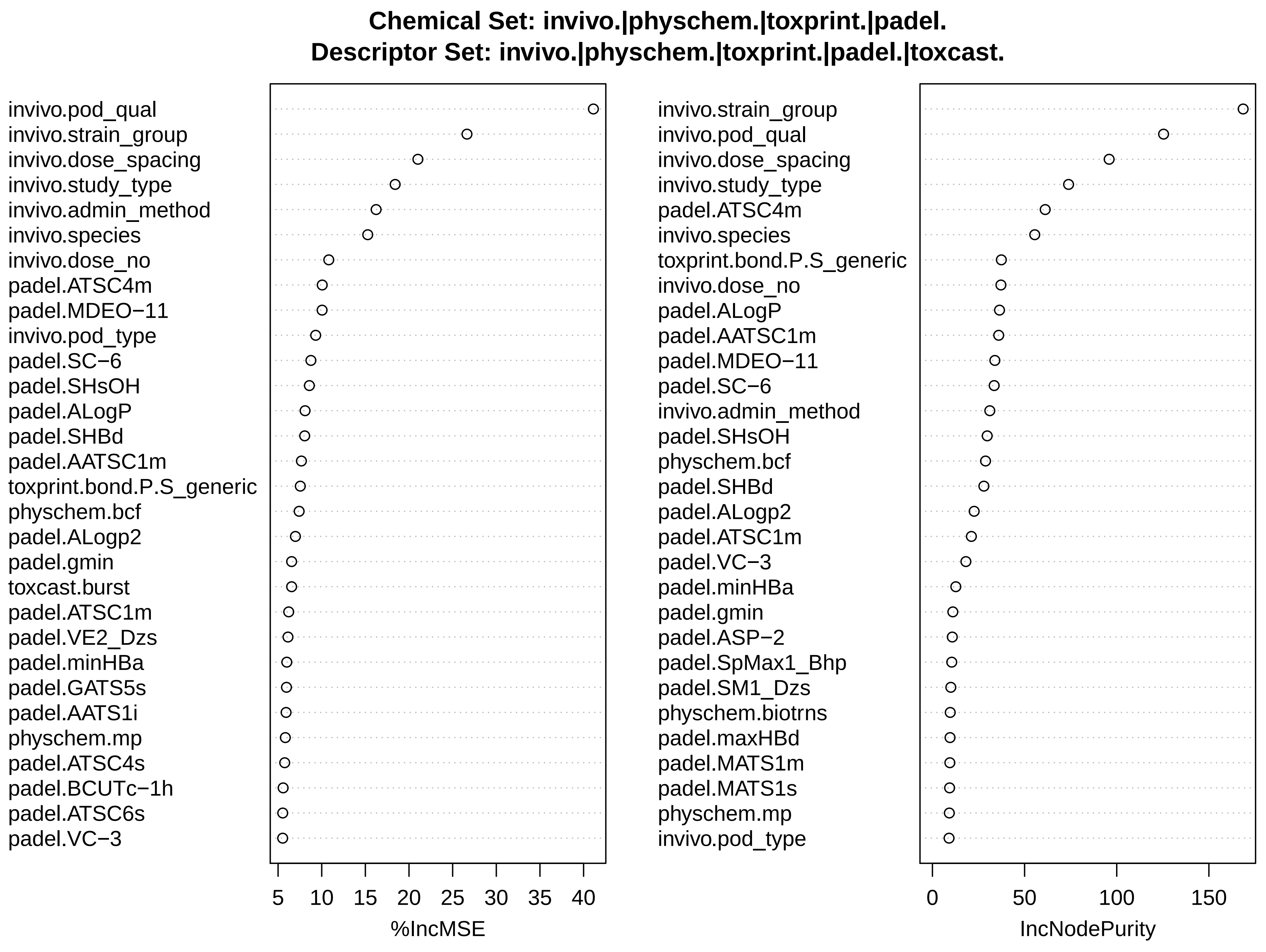
Chemical Set: invivo.|physchem.|toxprint.|padel.
Descriptor Set: invivo.|physchem.|toxprint.|padel.|toxcast.
invivo.pod_qual
invivo.strain_group
invivo.dose_spacing
invivo.study_type
invivo.admin_method
invivo.species
invivo.dose_no
padel.ATSC4m
padel.MDEO−11
invivo.pod_type
padel.SC−6
padel.SHsOH
padel.ALogP
padel.SHBd
padel.AATSC1m
toxprint.bond.P.S_generic
physchem.bcf
padel.ALogp2
padel.gmin
toxcast.burst
padel.ATSC1m
padel.VE2_Dzs
padel.minHBa
padel.GATS5s
padel.AATS1i
physchem.mp
padel.ATSC4s
padel.BCUTc−1h
padel.ATSC6s
padel.VC−3
5
10
15
20
25
30
35
40
%IncMSE
invivo.strain_group
invivo.pod_qual
invivo.dose_spacing
invivo.study_type
padel.ATSC4m
invivo.species
toxprint.bond.P.S_generic
invivo.dose_no
padel.ALogP
padel.AATSC1m
padel.MDEO−11
padel.SC−6
invivo.admin_method
padel.SHsOH
physchem.bcf
padel.SHBd
padel.ALogp2
padel.ATSC1m
padel.VC−3
padel.minHBa
padel.gmin
padel.ASP−2
padel.SpMax1_Bhp
padel.SM1_Dzs
physchem.biotrns
padel.maxHBd
padel.MATS1m
padel.MATS1s
physchem.mp
invivo.pod_type
0
50
100
150
IncNodePurity

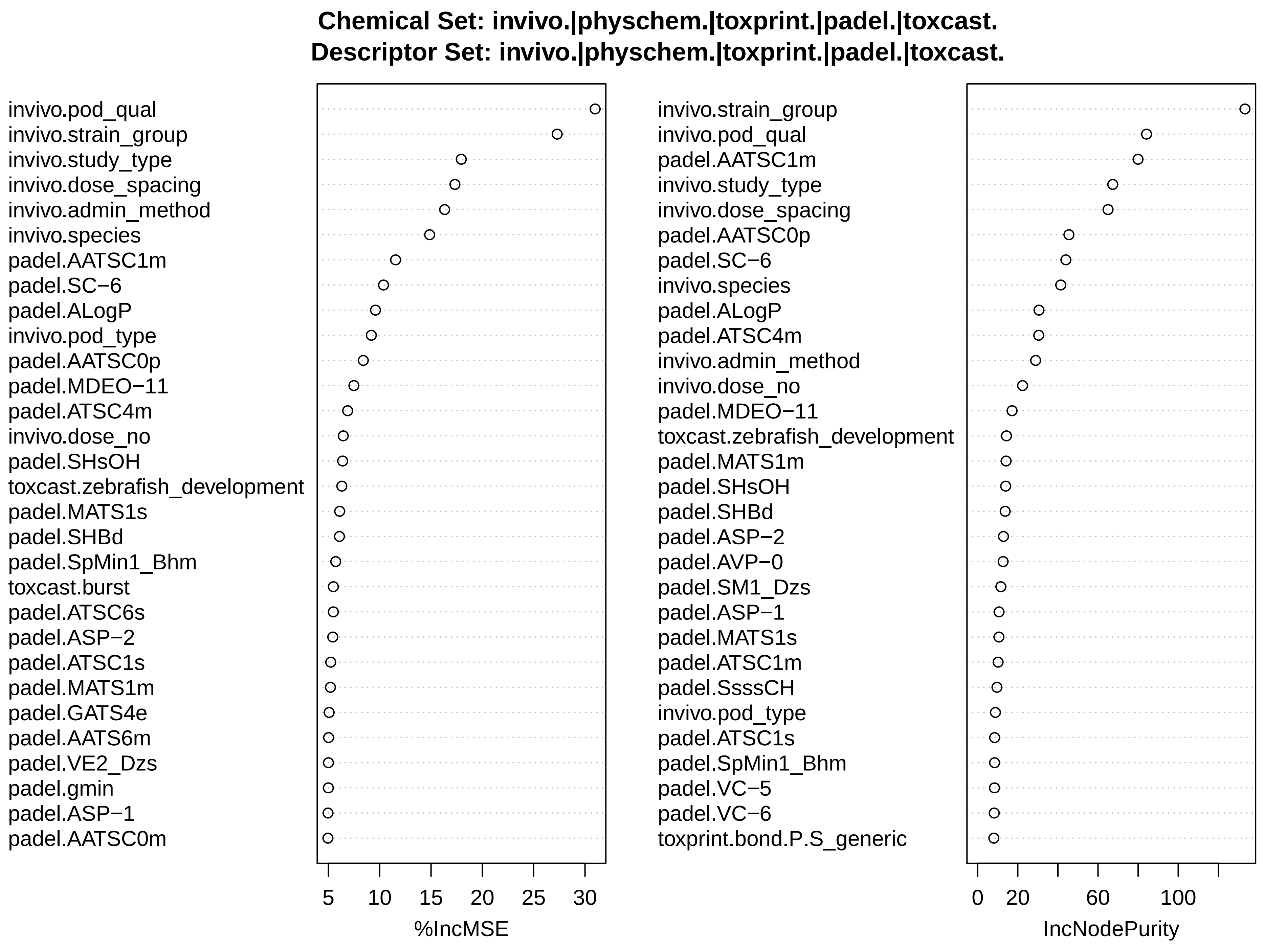
Chemical Set: invivo.|physchem.|toxprint.|padel.|toxcast.
Descriptor Set: invivo.|physchem.|toxprint.|padel.|toxcast.
invivo.pod_qual
invivo.strain_group
invivo.study_type
invivo.dose_spacing
invivo.admin_method
invivo.species
padel.AATSC1m
padel.SC−6
padel.ALogP
invivo.pod_type
padel.AATSC0p
padel.MDEO−11
padel.ATSC4m
invivo.dose_no
padel.SHsOH
toxcast.zebrafish_development
padel.MATS1s
padel.SHBd
padel.SpMin1_Bhm
toxcast.burst
padel.ATSC6s
padel.ASP−2
padel.ATSC1s
padel.MATS1m
padel.GATS4e
padel.AATS6m
padel.VE2_Dzs
padel.gmin
padel.ASP−1
padel.AATSC0m
5
10
15
20
25
30
%IncMSE
invivo.strain_group
invivo.pod_qual
padel.AATSC1m
invivo.study_type
invivo.dose_spacing
padel.AATSC0p
padel.SC−6
invivo.species
padel.ALogP
padel.ATSC4m
invivo.admin_method
invivo.dose_no
padel.MDEO−11
toxcast.zebrafish_development
padel.MATS1m
padel.SHsOH
padel.SHBd
padel.ASP−2
padel.AVP−0
padel.SM1_Dzs
padel.ASP−1
padel.MATS1s
padel.ATSC1m
padel.SsssCH
invivo.pod_type
padel.ATSC1s
padel.SpMin1_Bhm
padel.VC−5
padel.VC−6
toxprint.bond.P.S_generic
0
20
60
100
IncNodePurity

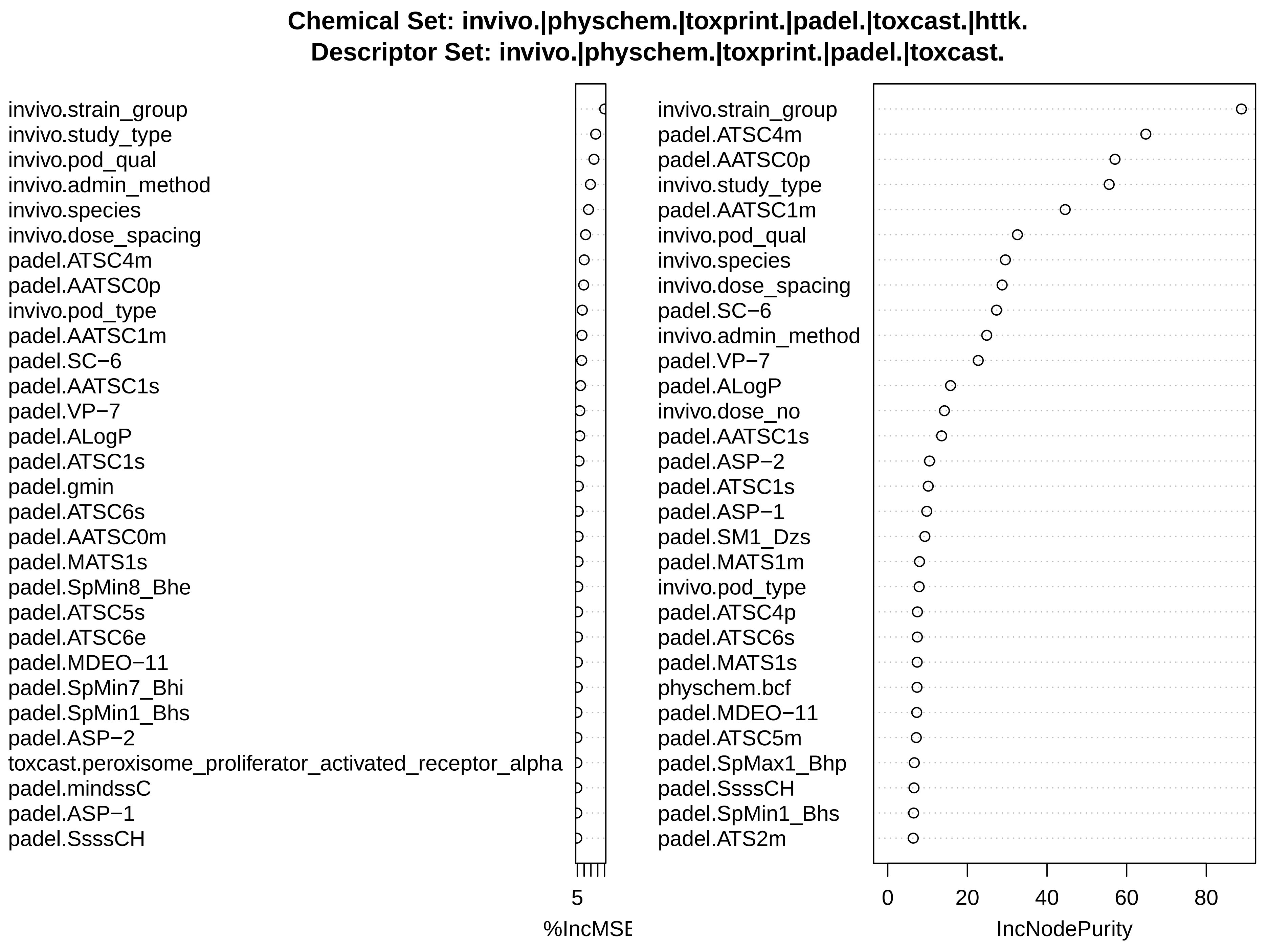

Chemical Set: invivo.|physchem.|toxprint.|padel.|toxcast.|httk.
Descriptor Set: invivo.|physchem.|toxprint.|padel.|toxcast.
invivo.strain_group
invivo.study_type
invivo.pod_qual
invivo.admin_method
invivo.species
invivo.dose_spacing
padel.ATSC4m
padel.AATSC0p
invivo.pod_type
padel.AATSC1m
padel.SC−6
padel.AATSC1s
padel.VP−7
padel.ALogP
padel.ATSC1s
padel.gmin
padel.ATSC6s
padel.AATSC0m
padel.MATS1s
padel.SpMin8_Bhe
padel.ATSC5s
padel.ATSC6e
padel.MDEO−11
padel.SpMin7_Bhi
padel.SpMin1_Bhs
padel.ASP−2
toxcast.peroxisome_proliferator_activated_receptor_alpha
padel.mindssC
padel.ASP−1
padel.SsssCH
5
%IncMSE
invivo.strain_group
padel.ATSC4m
padel.AATSC0p
invivo.study_type
padel.AATSC1m
invivo.pod_qual
invivo.species
invivo.dose_spacing
padel.SC−6
invivo.admin_method
padel.VP−7
padel.ALogP
invivo.dose_no
padel.AATSC1s
padel.ASP−2
padel.ATSC1s
padel.ASP−1
padel.SM1_Dzs
padel.MATS1m
invivo.pod_type
padel.ATSC4p
padel.ATSC6s
padel.MATS1s
physchem.bcf
padel.MDEO−11
padel.ATSC5m
padel.SpMax1_Bhp
padel.SsssCH
padel.SpMin1_Bhs
padel.ATS2m
0
20
40
60
80
IncNodePurity

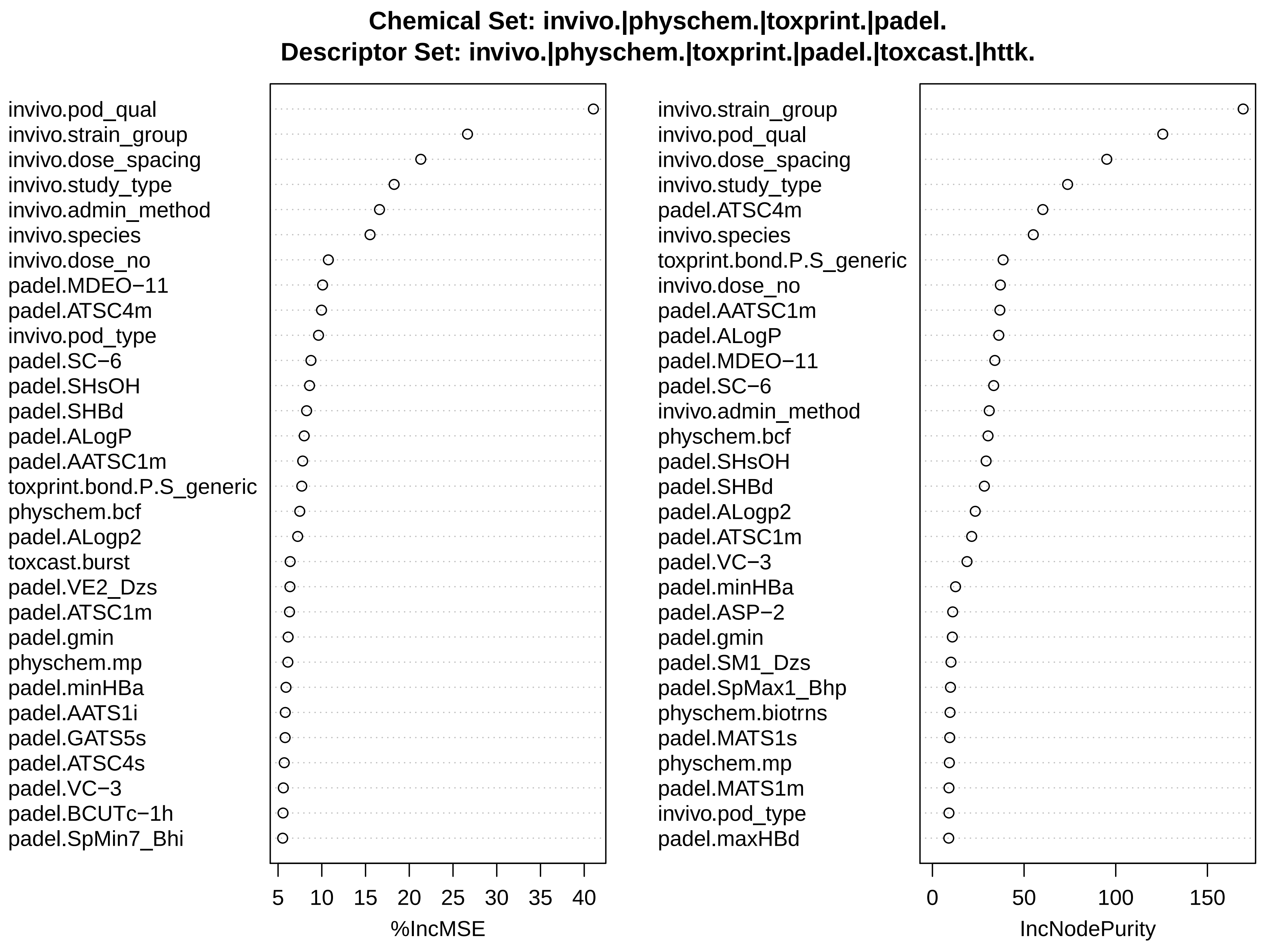
Chemical Set: invivo.|physchem.|toxprint.|padel.
Descriptor Set: invivo.|physchem.|toxprint.|padel.|toxcast.|httk.
invivo.pod_qual
invivo.strain_group
invivo.dose_spacing
invivo.study_type
invivo.admin_method
invivo.species
invivo.dose_no
padel.MDEO−11
padel.ATSC4m
invivo.pod_type
padel.SC−6
padel.SHsOH
padel.SHBd
padel.ALogP
padel.AATSC1m
toxprint.bond.P.S_generic
physchem.bcf
padel.ALogp2
toxcast.burst
padel.VE2_Dzs
padel.ATSC1m
padel.gmin
physchem.mp
padel.minHBa
padel.AATS1i
padel.GATS5s
padel.ATSC4s
padel.VC−3
padel.BCUTc−1h
padel.SpMin7_Bhi
5 10 15 20 25 30 35 40
%IncMSE
invivo.strain_group
invivo.pod_qual
invivo.dose_spacing
invivo.study_type
padel.ATSC4m
invivo.species
toxprint.bond.P.S_generic
invivo.dose_no
padel.AATSC1m
padel.ALogP
padel.MDEO−11
padel.SC−6
invivo.admin_method
physchem.bcf
padel.SHsOH
padel.SHBd
padel.ALogp2
padel.ATSC1m
padel.VC−3
padel.minHBa
padel.ASP−2
padel.gmin
padel.SM1_Dzs
padel.SpMax1_Bhp
physchem.biotrns
padel.MATS1s
physchem.mp
padel.MATS1m
invivo.pod_type
padel.maxHBd
0 50 100 150
IncNodePurity

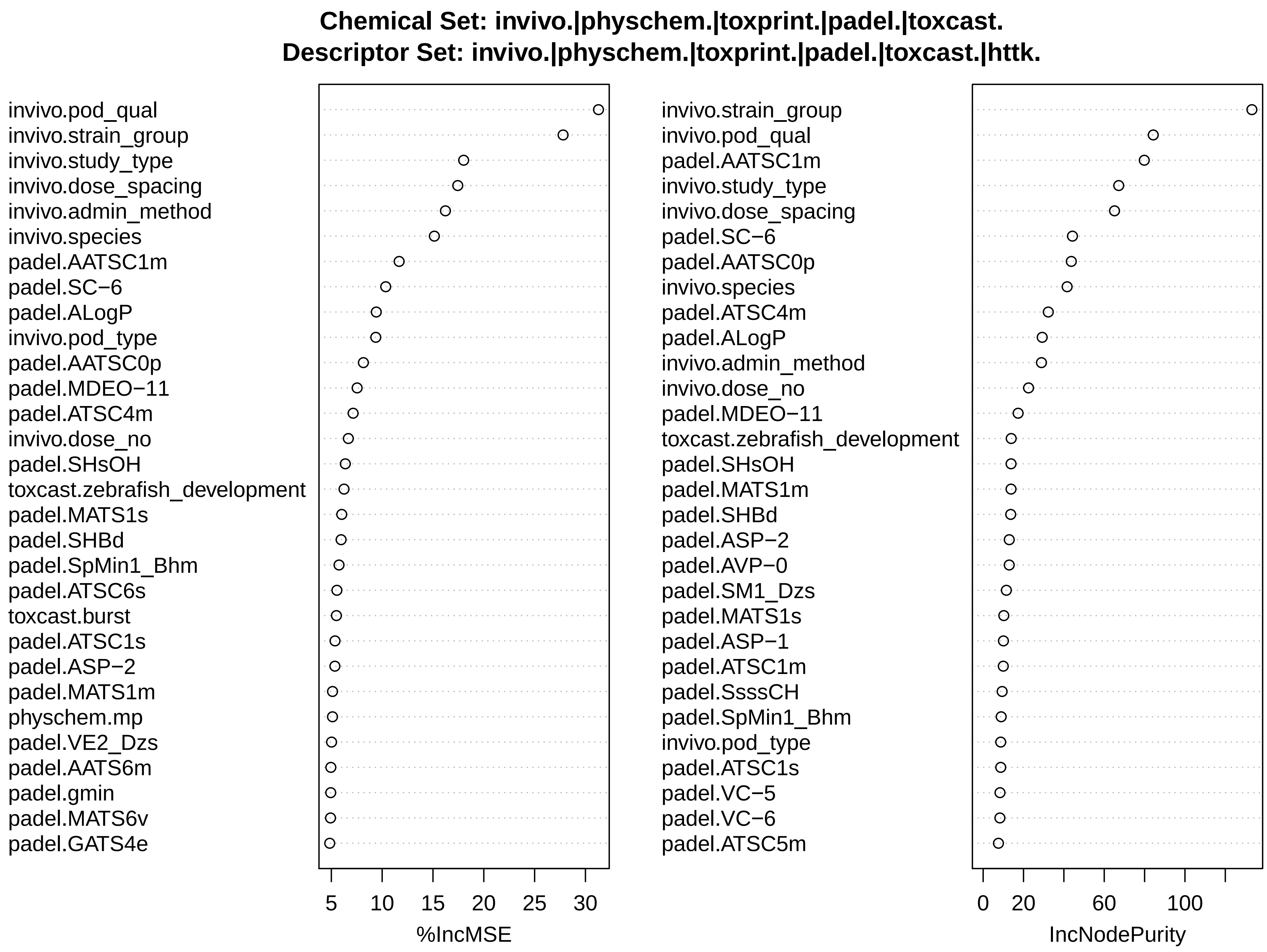
Chemical Set: invivo.|physchem.|toxprint.|padel.|toxcast.
Descriptor Set: invivo.|physchem.|toxprint.|padel.|toxcast.|httk.
invivo.pod_qual
invivo.strain_group
invivo.study_type
invivo.dose_spacing
invivo.admin_method
invivo.species
padel.AATSC1m
padel.SC−6
padel.ALogP
invivo.pod_type
padel.AATSC0p
padel.MDEO−11
padel.ATSC4m
invivo.dose_no
padel.SHsOH
toxcast.zebrafish_development
padel.MATS1s
padel.SHBd
padel.SpMin1_Bhm
padel.ATSC6s
toxcast.burst
padel.ATSC1s
padel.ASP−2
padel.MATS1m
physchem.mp
padel.VE2_Dzs
padel.AATS6m
padel.gmin
padel.MATS6v
padel.GATS4e
5
10
15
20
25
30
%IncMSE
invivo.strain_group
invivo.pod_qual
padel.AATSC1m
invivo.study_type
invivo.dose_spacing
padel.SC−6
padel.AATSC0p
invivo.species
padel.ATSC4m
padel.ALogP
invivo.admin_method
invivo.dose_no
padel.MDEO−11
toxcast.zebrafish_development
padel.SHsOH
padel.MATS1m
padel.SHBd
padel.ASP−2
padel.AVP−0
padel.SM1_Dzs
padel.MATS1s
padel.ASP−1
padel.ATSC1m
padel.SsssCH
padel.SpMin1_Bhm
invivo.pod_type
padel.ATSC1s
padel.VC−5
padel.VC−6
padel.ATSC5m
0
20
60
100
IncNodePurity

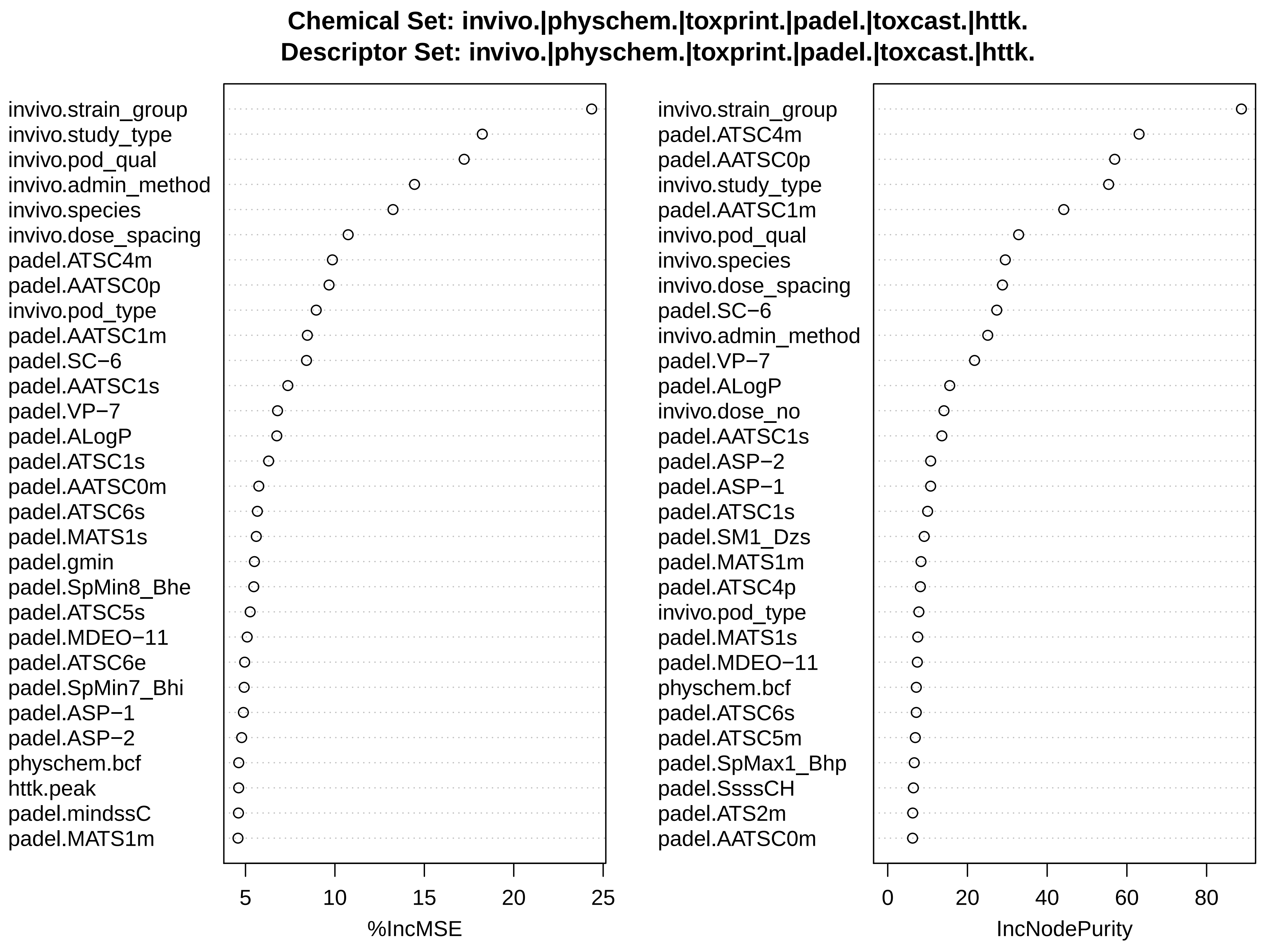
Chemical Set: invivo.|physchem.|toxprint.|padel.|toxcast.|httk.
Descriptor Set: invivo.|physchem.|toxprint.|padel.|toxcast.|httk.
invivo.strain_group
invivo.study_type
invivo.pod_qual
invivo.admin_method
invivo.species
invivo.dose_spacing
padel.ATSC4m
padel.AATSC0p
invivo.pod_type
padel.AATSC1m
padel.SC−6
padel.AATSC1s
padel.VP−7
padel.ALogP
padel.ATSC1s
padel.AATSC0m
padel.ATSC6s
padel.MATS1s
padel.gmin
padel.SpMin8_Bhe
padel.ATSC5s
padel.MDEO−11
padel.ATSC6e
padel.SpMin7_Bhi
padel.ASP−1
padel.ASP−2
physchem.bcf
httk.peak
padel.mindssC
padel.MATS1m
5
10
15
20
25
%IncMSE
invivo.strain_group
padel.ATSC4m
padel.AATSC0p
invivo.study_type
padel.AATSC1m
invivo.pod_qual
invivo.species
invivo.dose_spacing
padel.SC−6
invivo.admin_method
padel.VP−7
padel.ALogP
invivo.dose_no
padel.AATSC1s
padel.ASP−2
padel.ASP−1
padel.ATSC1s
padel.SM1_Dzs
padel.MATS1m
padel.ATSC4p
invivo.pod_type
padel.MATS1s
padel.MDEO−11
physchem.bcf
padel.ATSC6s
padel.ATSC5m
padel.SpMax1_Bhp
padel.SsssCH
padel.ATS2m
padel.AATSC0m
0
20
40
60
80
IncNodePurity

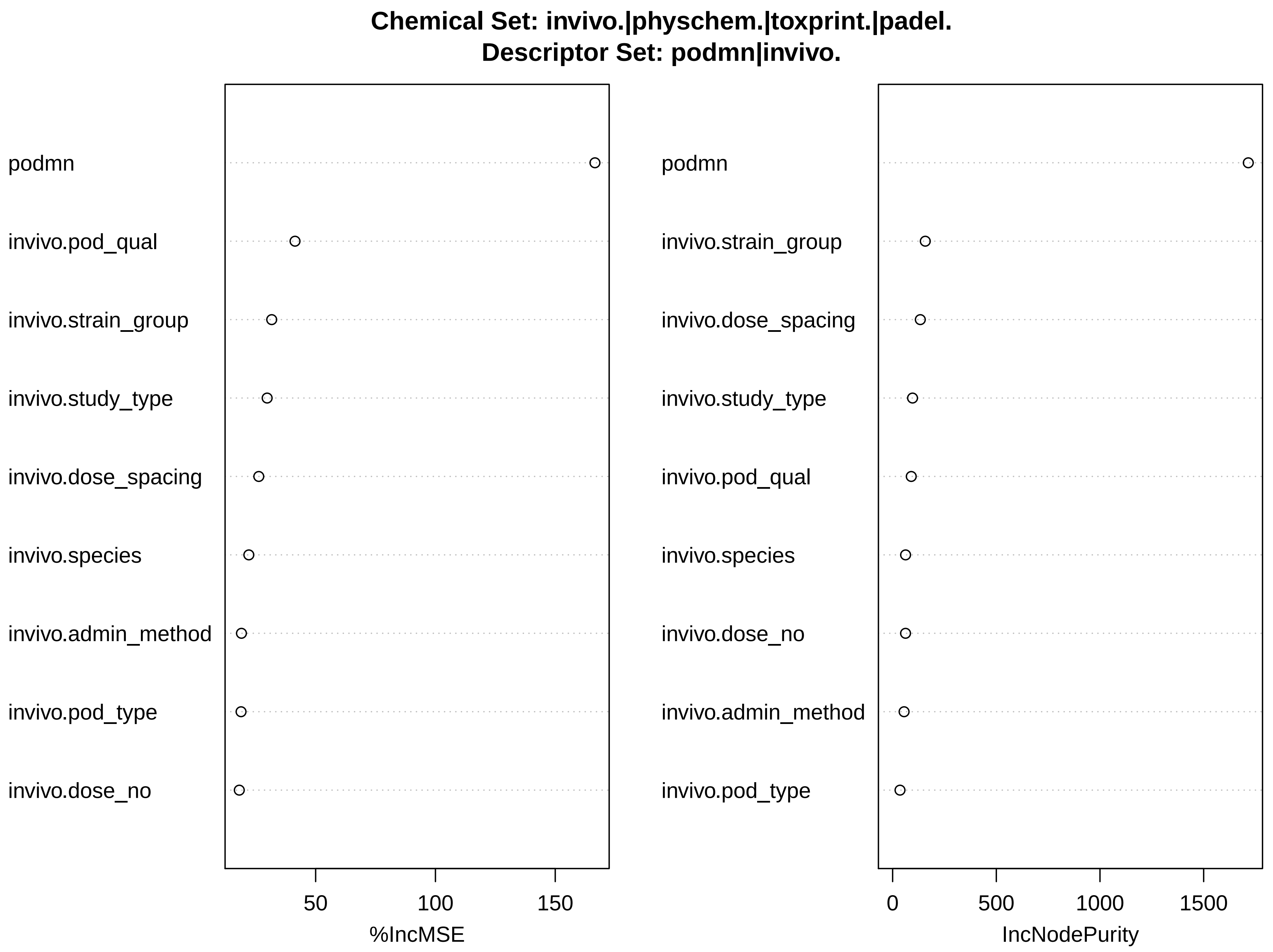
Chemical Set: invivo.|physchem.|toxprint.|padel.
Descriptor Set: podmn|invivo.
podmn
invivo.pod_qual
invivo.strain_group
invivo.study_type
invivo.dose_spacing
invivo.species
invivo.admin_method
invivo.pod_type
invivo.dose_no
50
100
150
%IncMSE
podmn
invivo.strain_group
invivo.dose_spacing
invivo.study_type
invivo.pod_qual
invivo.species
invivo.dose_no
invivo.admin_method
invivo.pod_type
0
500
1000
1500
IncNodePurity

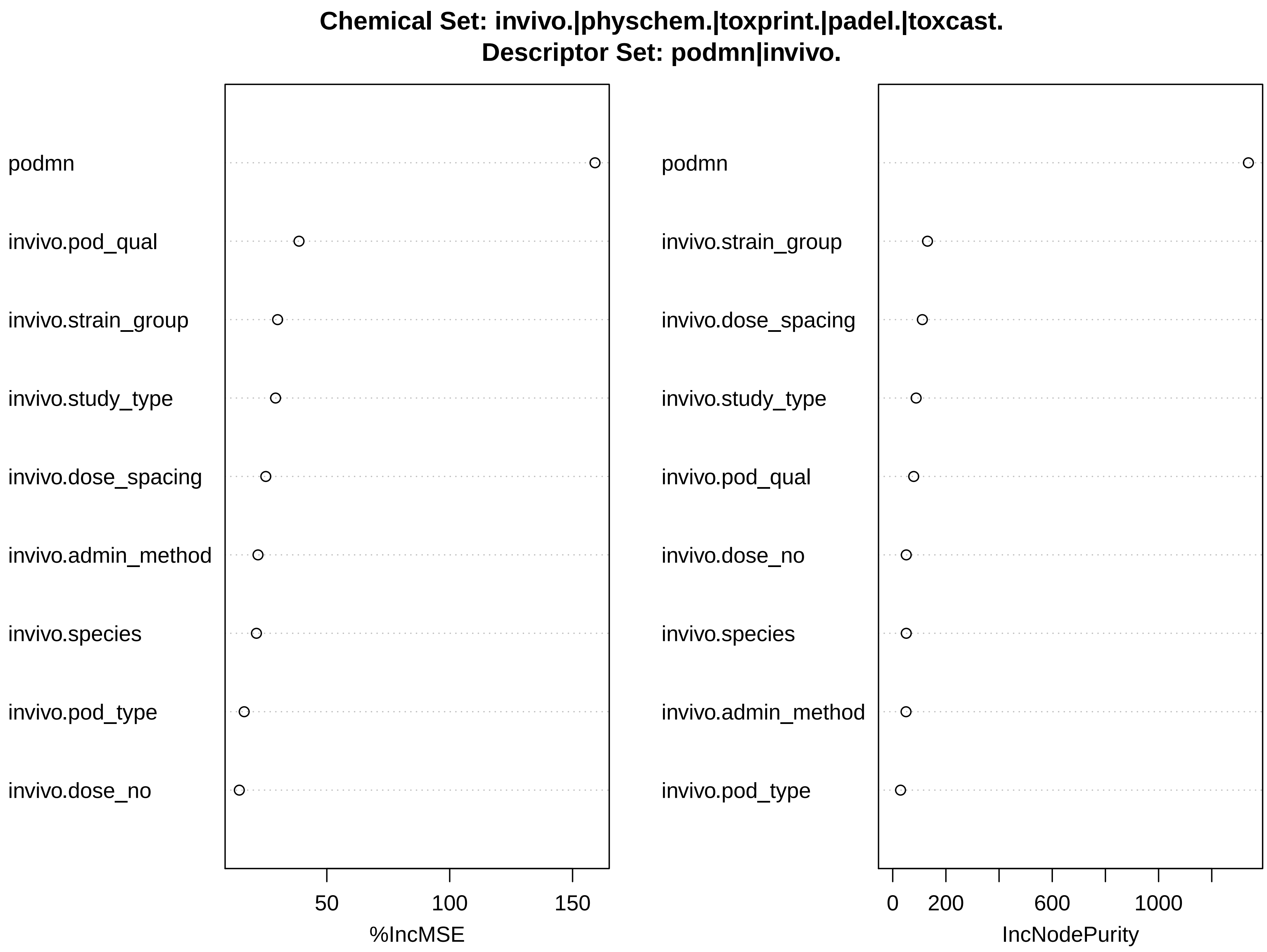
Chemical Set: invivo.|physchem.|toxprint.|padel.|toxcast.
Descriptor Set: podmn|invivo.
podmn
invivo.pod_qual
invivo.strain_group
invivo.study_type
invivo.dose_spacing
invivo.admin_method
invivo.species
invivo.pod_type
invivo.dose_no
50
100
150
%IncMSE
podmn
invivo.strain_group
invivo.dose_spacing
invivo.study_type
invivo.pod_qual
invivo.dose_no
invivo.species
invivo.admin_method
invivo.pod_type
0
200
600
1000
IncNodePurity

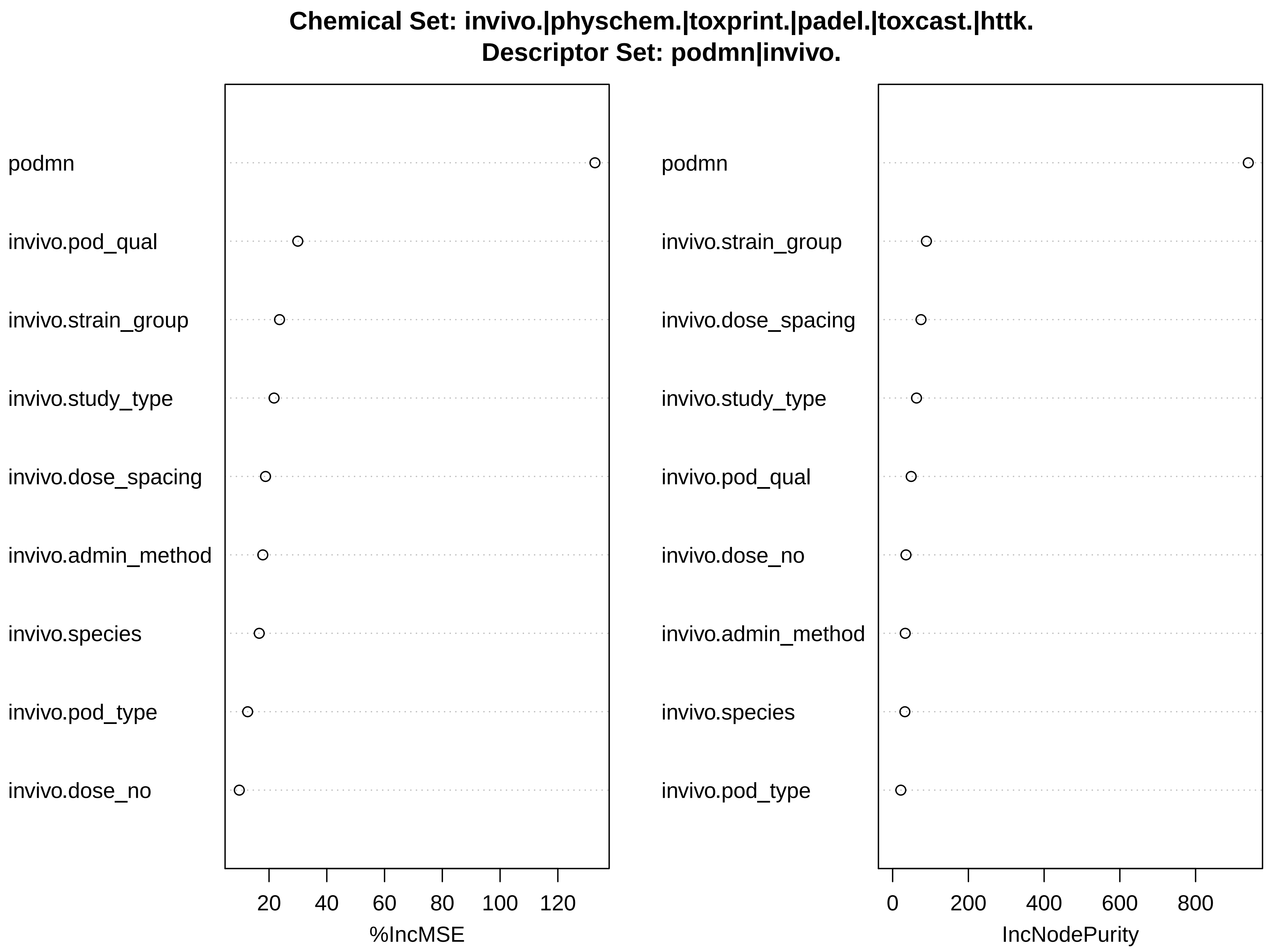

Chemical Set: invivo.|physchem.|toxprint.|padel.|toxcast.|httk.
Descriptor Set: podmn|invivo.
podmn
invivo.pod_qual
invivo.strain_group
invivo.study_type
invivo.dose_spacing
invivo.admin_method
invivo.species
invivo.pod_type
invivo.dose_no
20
40
60
80
100
120
%IncMSE
podmn
invivo.strain_group
invivo.dose_spacing
invivo.study_type
invivo.pod_qual
invivo.dose_no
invivo.admin_method
invivo.species
invivo.pod_type
0
200
400
600
800
IncNodePurity
